# Neurofilaments in motor neuron disorders: towards promising diagnostic and prognostic biomarkers

**DOI:** 10.1186/s13024-020-00406-3

**Published:** 2020-10-15

**Authors:** Elisabetta Zucchi, Valentina Bonetto, Gianni Sorarù, Ilaria Martinelli, Piero Parchi, Rocco Liguori, Jessica Mandrioli

**Affiliations:** 1 Department of Biomedical, Metabolic and Neural Science, University of Modena and Reggio Emilia, Modena, Italy; 2 Department of Biochemistry and Molecular Pharmacology, Istituto di Ricerche Farmacologiche Mario Negri IRCCS, Milan, Italy; 3 Neuromuscular Center, Department of Neurosciences, University of Padova, Padua, Italy; 4 Clinica Neurologica, Azienda Ospedaliera di Padova, Padua, Italy; 5 Department of Neurosciences, Azienda Ospedaliero Universitaria Modena, Modena, Italy; 6 IRCCS Istituto delle Scienze Neurologiche, Ospedale Bellaria, Bologna, Italy; 7 Department of Experimental, Diagnostic and Specialty Medicine, University of Bologna, Bologna, Italy; 8 Department of Biomedical and Neuromotor Sciences, University of Bologna, Bologna, Italy

**Keywords:** Amyotrophic lateral sclerosis, Motor neuron disorder, Neurofilament, Biomarkers, Diagnostic, Prognostic, Disease-progression, Sensitivity

## Abstract

Motor neuron diseases (MNDs) are etiologically and biologically heterogeneous diseases. The pathobiology of motor neuron degeneration is still largely unknown, and no effective therapy is available. Heterogeneity and lack of specific disease biomarkers have been appointed as leading reasons for past clinical trial failure, and biomarker discovery is pivotal in today’s MND research agenda.

In the last decade, neurofilaments (NFs) have emerged as promising biomarkers for the clinical assessment of neurodegeneration. NFs are scaffolding proteins with predominant structural functions contributing to the axonal cytoskeleton of myelinated axons. NFs are released in CSF and peripheral blood as a consequence of axonal degeneration, irrespective of the primary causal event. Due to the current availability of highly-sensitive automated technologies capable of precisely quantify proteins in biofluids in the femtomolar range, it is now possible to reliably measure NFs not only in CSF but also in blood.

In this review, we will discuss how NFs are impacting research and clinical management in ALS and other MNDs. Besides contributing to the diagnosis at early stages by differentiating between MNDs with different clinical evolution and severity, NFs may provide a useful tool for the early enrolment of patients in clinical trials. Due to their stability across the disease, NFs convey prognostic information and, on a larger scale, help to stratify patients in homogenous groups. Shortcomings of NFs assessment in biofluids will also be discussed according to the available literature in the attempt to predict the most appropriate use of the biomarker in the MND clinic.

## Background

Amyotrophic Lateral Sclerosis (ALS), the most prevalent among motor neuron disorders (MNDs), usually affects the motor system at both central and peripheral levels. However, the disease may also selectively or differentially involve the upper or lower motor neurons (respectively UMN and LMN), and may even include an extra-motor dimension, leading to behavioural and cognitive dysfunction [1–3]. Expanded knowledge of the genotypic and phenotypic variability of the disease suggests the possibility of different pathogenic trajectories, which would explain, for example, the existence of certain extremes within MND spectrum with selective UMN or LMN involvement and slower progression, such as primary lateral sclerosis (PLS) or progressive muscular atrophy (PMA). The findings of peripheral nerve involvement in post-mortem PLS [4], and of corticospinal tract degeneration with ubiquitin inclusions in half of the cases of PMA [5], seem to confirm that these diseases belong to the same spectrum as ALS. Additionally, discrete ALS phenotypes such as flail arm or flail leg demonstrate a slower disease course compared to classic “Charcot”-like forms [6]. Large-scale whole-exome sequencing studies showed that the age of onset or survival in ALS patients might be influenced by several genetic variants [7, 8]. A general assumption is that the disease results from a complex interplay between genetic and environmental factors, with involved genes clustering into three major categories, namely protein homeostasis, RNA homeostasis/processing, and cytoskeletal dynamics, and immune dysregulation having a major role in determining disease onset and progression [9, 10]. Accordingly, proteomic alterations in circulating monocytes were associated with an earlier disease onset [11], pro-inflammatory cytokines were shown to aggravate and accelerate disease progression [12–14], while T-reg cell dysfunction correlated with disease progression and survival [15, 16].

Overall, ALS can be defined as an “etiologically and biologically heterogeneous disease” [EVID] [17, 18] associated with extreme phenotypic variability, a feature thought to have significantly contributed to the failure of the experimental drug trials conducted to date [19, 20]. Investigating biomarkers in MNDs may, therefore, have several aims and applications, from a deeper understanding of the pathological basis of the disease to implications in the clinical practice, allowing for anticipated planning of due interventions. Furthermore, appropriate stratification of patients according to biomarker values, may implement trial design and favour research for disease treatments. For this reason, the search for biomarkers has been incorporated into Airlie House consensus guidelines for trial implementation [20]. In these consensus criteria, particular attention was drawn on the necessity to include prognostic and predictive biomarkers as eligibility criteria, and pharmacodynamics biomarkers as a proof of the adequacy of drug delivery, target engagement, or biological activity of the experimental therapeutic. One of the contributing factors to the failure of previous clinical trials was the inadequate statistical power, with highly promising phase II studies missing the primary endpoints in the broader phase III trials. The use of known clinical variables for patients’ stratification requires large numbers of patients with prolonged clinical follow-up. Validated prognostic biomarkers may aid in the identification of which subset of patients have a higher likelihood of demonstrating the effect of the experimental therapeutic, shortening the duration of follow-up and the necessity of broad recruitments. Thus, the pathway for biomarker discovery and validation in ALS is tightly connected to that of therapeutic development and pathogenic characterization. Therefore, several biomarker types, as defined by BEST glossary [21], would have a wide range of applications in ALS.

In the last decade, neurofilaments (NFs), a structural component of the axonal cytoskeleton, have emerged as a rather unspecific but extremely sensitive biomarker of neurodegeneration across many neurological diseases [22, 23]. This review will focus on the potential roles of NFs in the MND biomarker platform discovery, with a particular emphasis on the advances brought by their potential use in clinical practice and trial optimization. Next, their shortcomings will also be discussed, highlighting the missing steps for their validation pathway and the intrinsic limitations that should be overcome by other biomarkers.

## Main text

### NF structure and composition

NFs are cytoskeletal structures composed of 10 nm large filaments (10 nm), with a diameter intermediate between actin (6.5 nm) and microtubules (25 nm), belonging to the class of intermediate filaments. NFs are structural proteins of neurons, which assemble with alpha-internexin in the central nervous system (CSN) and peripherin in the peripheral nervous system (PNS). Three NF isoforms are recognized, which are named according to their molecular weight (neurofilament light chain, NfL; neurofilament medium chain, NfM; neurofilament heavy chain, NfH). Their relative molecular masses, comprising post-translational modifications, are, respectively, 61.5 kDa and 70–86 kDa; 102.5 kDa and 145–160 kDa; 112.5 kDa and 200–220 kDa), as detected by sodium dodecyl sulphate (SDS) polyacrylamide gel electrophoresis. These isoforms share a structure with a relatively conserved central α-helical rod region, a short variable head domain at the amino-terminal end, and a variably long tail at the carboxy-terminus [24]. The NF proteins assemble through their hydrophobic central region forming coil-to-coil dimers and subsequently heteropolymers in cylindrical unit-length filament structures, which elongate by annealing, and are radially compacted to form 10 nm large fibrils [25]. NF functions include a purely structural role in the axonal cytoskeleton, the transport and docking of organelles such as mitochondria and endoplasmic reticulum, and the participation in intracellular signalling and transcription [26]. Moreover, they are involved in the regulation of the radial growth of large fast-conducting neurons [24, 27]. A salient step in the cellular processing of NF isoforms is represented by post-translational modifications, mainly phosphorylation, O-linked glycosylation, nitration, oxidation, and ubiquitylation. Phosphorylation most frequently occurs in the three subunits of the head domain, as well as in the tail domains, especially in NfM and NfH, given their abundance in lysine-serine-proline (KSP) repeat domains. Phosphorylation, other than conferring resistance to proteases [28], contributes to generate the neurofibrillary structure of the protein. Indeed, phosphorylation of the head domain regulates NF polymerization, whereas that of the tail mediates the spacing between NF polymers in both NfM and NfH [29, 30]. These processes are thought to occur in sequential steps in different regions of the neurons, with the head domain being phosphorylated in the cell body, and the tail domain only after the protein reach the axon [29].

Interestingly, NFs form a liquid crystal gel network in a variety of neurodegenerative diseases, including ALS, dementia with Lewy bodies (DLB), and Parkinson’s disease (PD) [28, 29]. In ALS, accumulation of bundled NF in axonal spheroids is accounted as one histopathological hallmark, together with hyperphosphorylation of NfH and NfM in the tail domains and the presence of NF proteins in perikaryal inclusions [31–34]. Though the mechanism of aggregation is not yet fully elucidated, it seems to be mediated by hyperphosphorylation phenomena [35], which can affect the stoichiometry of NF composition, rendering them more prone to aggregation [36, 37]. Interestingly, alterations in NF stoichiometry have been reproduced in animal models by overexpressing one subunit over the others, resulting in axonal swellings and progressive neuropathic changes highly reminiscent of ALS pathological changes [38, 39]. On the other hand, modifications in NF stoichiometry might also be an adaptive strategy to save energy in the degenerating motor neuron, as other studies suggest [40]. Further longitudinal studies on NF stoichiometry from the presymptomatic phase to the onset and progression of the disease in animal models and human subjects carrying different disease associated gene-mutations would be helpful to untangle these structural aspects.

**Fig. 1:**
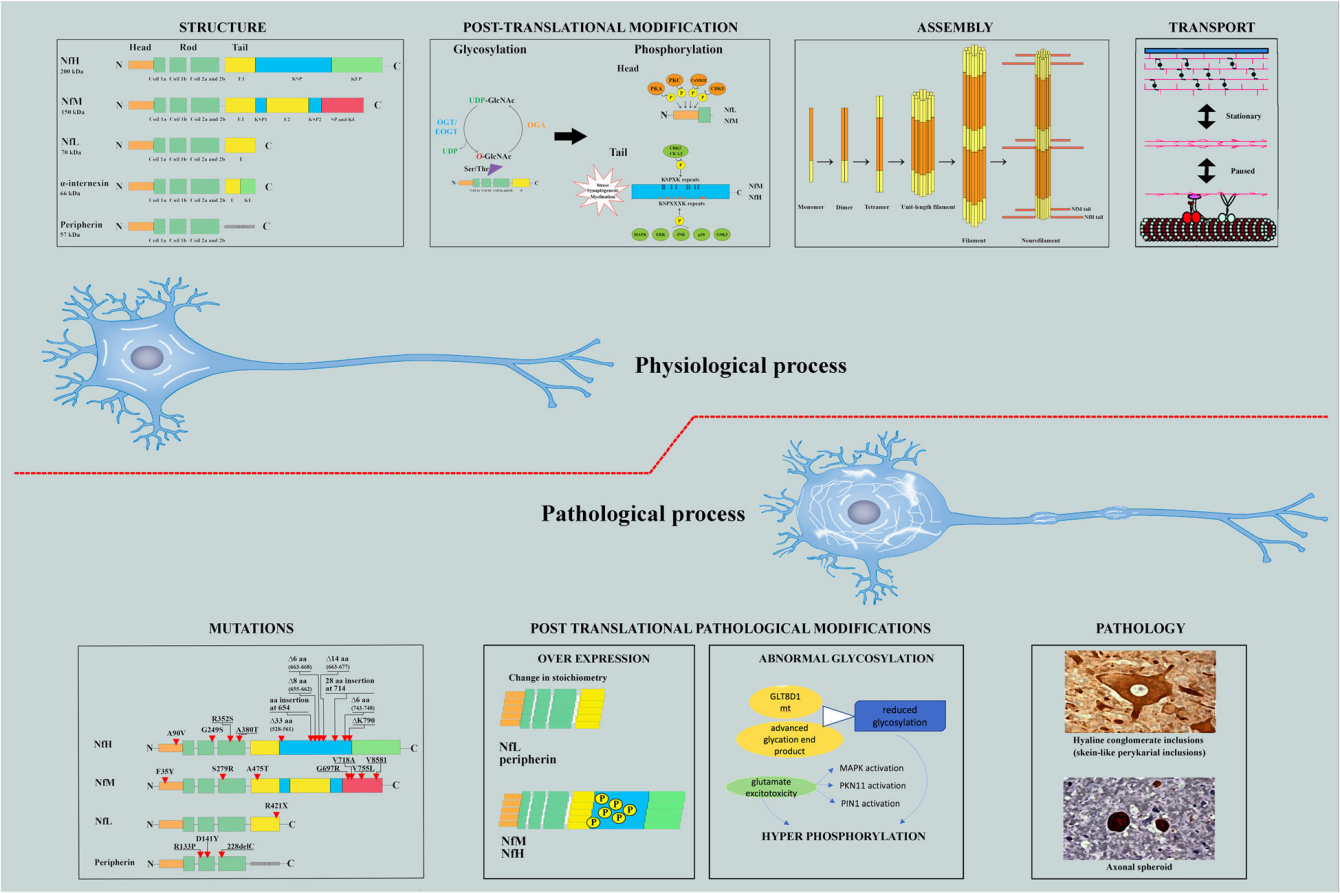
NFs structure, physiological processes and pathological modifications in ALS. The cartoon’s upper panel shows the physiological structure and composition of NF isoforms; note the common structure with a variable N-terminal head and the conserved central coiled coil across all isoforms on the left window. The variably elongated C-terminal tail domain, particularly represented in NfM and NfH, is rich in KSP repeats, which are sites for post-translational phosphorylation. Main post-translational modifications (central window), include glycosylation through the attachment of O-linked N-acetylglucosamine (O-GlcNAc) to individual Ser and Thr residues by O-linked N-acetylglucosamine transferase (OGT) and phosphorylation, which are reciprocal processes, i.e., reduced glycosylation may result in excess harmful phosphorylation [41]. Phosphorylation results from the tightly regulated activity of several kinases and phosphatases commanded by second-messenger pathways. The head domain of every NF isoform is phosphorylated in the cell body by protein kinase A (PKA), protein kinase C (PKC), calcium/calmodulin-dependent protein kinase type II (CAMKII) and CDK5 [24]. In contrast, phosphorylation in the tail KSP repeats almost exclusively occurs in NfM, and NfH on their entry into and transit along the axon. This last process is highly dynamic and seems to be involved in the axonal transport of NF during myelination or synaptogenesis; however, it may also be triggered by stress phenomena. The pairing of parallel heterodimers and antiparallel tetramers, by interactions between coil domains, mediates the assembly of NF isoforms; eight tetramers associate to form cylindrical structures known as unit-length filaments (ULFs), which approximate the final diameter of NF. End-to-end annealing of ULFs allows longitudinal elongation, while radial compaction results in the final width of 10 um in the mature NF polymer. The net forward movement of NF along the axons (right upper window) is the result of a rapid intermittent phase driven by molecular motors (dynein and dynactin) carrying hetero-oligomers along the microtubule and pauses which are determined by reversible attachment of NFs. However, the majority of NF isoforms resides in a stable network, representing the stationary phase of NF transport along the axon [24]. Molecular aberrations within these physiological processes may result in motor neuron pathologies, as shown in the lower panel. In the first window on the left, red triangles highlight the mutations in NF isoforms known to cause ALS. Modifications in subunit stoichiometry by overexpression of one isoform over the others may result in pathological aggregates. For example, overexpression of peripherin or mutated NfL can cause ALS-like neurofilament aggregates and selective degeneration of spinal motor neurons, and NfM and NfH dysregulated upregulation, often accompanied by excessive phosphorylation, may result in aberrant perikaryal neurofilament aggregates [42]. Excessive phosphorylation may be promoted by reduced glycosylation activity due to a loss-of-function mutation in a glycosyltransferase, GLT8D1 [43]. Glutamate excitotoxicity activates several kinases (MAPK,PKN11, PIN1) which end up in NfM and NfH tail hyperphophorylation. This last process has been repeatedly associated with two major histopathological findings in ALS, namely hyaline conglomerate inclusions (HCIs), ubiquitin-negative floccular inclusions rich in neurofilaments and peripherin, and axonal spheroids, which are non-pathognomonic for ALS but seems to occur early during motor neuron degeneration in fALS and sALS (right lower window). Studies on hyperphosphorylation of NfM and NfH showed that this phenomenon may lead to a slowing of NF transport along the axon, with accumulation of Nf inclusions outside the nucleus and in the axon, engorgement of perikaryal structures and disrupted dynamics of axonal circuitry [44]. Abbreviations: NfL, neurofilament light chain; NfM, neurofilament medium chain; NfH, neurofilament heavy chain; E segment, glutamic-acid-rich segment; E1, glutamic-acid rich segment 1; E2, glutamic-acid-rich segment 2; KE, lysine–glutamic acid; KEP, lysine–glutamic acid–proline; SP, serine–proline; KSP, lysine–serine–proline; O-GlcNAc, O-linked N-acetylglucosamine; OGT, intracellular glycosyltransferase O-linked N-acetylglucosamine transferase; EOGT, extracellular glycosyltransferase EGF domain-specific O-linked N-acetylglucosamine transferase; OGA, O-GlcNAcase; PKA, protein kinase A; PKC, protein kinase C; CAMKII, calcium/calmodulin-dependent protein kinase type II; CDK5, cyclin-dependent kinase-5; MAPK, mitogen-activated protein kinase; ERK, extracellular signal-regulated kinase; JNK, c-Jun N-terminal kinases; GSK-3, Glycogen synthase kinase 3;GLT8D1, glycosyltransferase 8 domain-containing 1; PKN11, Serine/threonine protein kinase C-related kinase; PIN1, Peptidyl-prolyl cis-trans isomerase NIMA-interacting 1

Figure 1 summarizes key concepts around NF structure and physiology, highlighting current knowledge on pathological processes involving neurofilaments in MND.

### Technical advances in NF quantification in ALS

The intuition that purely neuronal proteins such as NFs might be elevated in extracellular fluids in neurodegenerative diseases as a result of the release from apoptotic cells or as part of the cell remodelling process dates back 1996 when Rosengren originally reported that CSF NfL levels are increased in patients with ALS and other neurological diseases [45]. Since then, methodological advances have significantly improved the detection and quantification of NFs. As stressed by Khalil et al. [22], four generations of assays can be counted at the moment, the first being represented by semi-quantitative Western blots, originally used to prove the increased CSF NFs values in neurological diseases compared to healthy controls. A move forward was then provided by the introduction of sandwich ELISA assays, which allowed the quantification of NFs in CSF, although their limit of detection was still too high to measure NFs plasma and serum reliably. Discrimination between ALS and healthy control samples was not always straightforward, especially for pNfH, as highlighted by a 2016 metanalysis [46]. However, also the reported mean concentration of NfL varied significantly, depending on the ELISA kit [47]. Furthermore, significant inter-laboratory variations occurred using the same kit, mostly because of the lack of accurate and consistent protein standards [48]. Pre-analytical variables also had a negative impact especially for pNfH quantification, requiring in some cases the protein denaturation to overcome aggregation phenomena [49]. The introduction of Electrochemolumiscence (ECL)-based ELISA kits further improved the analytical sensitivity of the assay for NfL and NfH quantification, allowing a reduction of the sample volume needed for the analysis [50, 51]. Lastly, the development of the single-molecule array (SiMoA) technology, which enhanced the sensitivity for the antigen of interest up to 126-fold and 25-fold when compared to the ELISA and ECL assays, respectively [52] provided the most significant methodological advance. Being SiMoA a fully automated procedure, the sources of error and the inter-operator and intra-assay variability are dramatically reduced warranting a higher reproducibility. While for NfM this technique has not flourished yet, several studies have explored SiMoA for quantifying NfL and NfH with success in a broad variety of conditions, proving its outstanding superiority to the other immunoassays [52, 53], in particular for the measurement of NFs in vivo in more accessible biofluids.

CSF is the closest fluid to neurons, collecting all molecules and signals motor neurons are sending in the extracellular space. Moreover, it is a low complexity fluid with small concentrations of cells and finely controlled concentrations of other solutes, resulting in a low interference with quantification by immunoassays. However, collecting CSF requires a lumbar tap, which is an invasive procedure, and repeated sampling is not part of the standard clinical practice, and may be difficult in ALS patients, where motility may decrease over time. Blood is a more accessible biofluid to look at for biomarker discovery, though concentrations of neuronal proteins might be many units lower than in CSF. Enhanced sensitivity of immunoassays allows nowadays the determination of peripheral NF levels in healthy subjects, thus establishing normative values. Thanks to the acquisition of these data on NFs, we know that CSF NfL increases physiologically with age at a rate of 3.30% per year starting from 20 years old, and that men display higher levels than women [54]. These observations, however, were not replicated in progressive neurodegenerative conditions, including ALS [54]. Recent studies also demonstrated a significant, albeit not full, correlations between NF concentrations in serum or plasma and CSF, implying that what is quantified at the periphery reliably represents what is happening within the CNS [55–61].

Holding these technical aspects of NF quantification, next we will discuss their potential clinical applications in the field of MNDs.

### NFs as diagnostic biomarkers in ALS and other MNDs

As it is usually the case for any novel biomarker, the first studies on NFs in MNDs attempted to determine the diagnostic power of these molecules. NfL detection in CSF distinguished ALS from healthy controls based on a 5 to 10 folds increased concentration of the protein in the former group [45]. Since then, several studies on both NfL and NfH in CSF confirmed the good diagnostic of these biomarkers in distinguishing ALS from healthy controls or ALS mimics [56, 62–67]. Although serum or plasma initially proved to perform significantly worse than CSF in discriminating ALS from healthy controls [46], a more recent study by SiMoA allowed the distinction of ALS patients from healthy controls and other neurodegenerative diseases such as Alzheimer’s or Parkinson’s diseases with an 85.5% sensitivity and an 81.8% specificity [68]. In a single study [69], the direct comparison of the performance between NfL and NfH in CSF revealed a better specificity for the latter, a finding which has never been replicated with other assays, including the SiMOA technology.

ALS mimic disorders, which initially could not be reliably differentiated from ALS, can now be identified with a significant degree of confidence [57, 58, 67, 69] In the largest case series of MND mimics reported to date, the highest levels of NFs involved pathologies such as CIDP, myopathies, and polyneuropathies [57, 64]. For a more detailed overview of studies analysing differential specificities of NFs in serum and CSF see Table 1. In contrast, slowly progressing MNDs such as hereditary spastic paraplegia [73, 74], spinal and bulbar muscular atrophy [75], and, possibly, PLS [74] showed close to normal levels of NFs, a finding which can help to rule out ominous conditions such as ALS. Though promising, these results require an inter-laboratory validation and their confirmation in larger patient cohorts. Moreover, the relatively long mean time-lapses occurring between the onset of motor symptoms and sampling due to the delay patients reach medical attention must also be addressed, given the lack of longitudinal studies answering the critical question of whether and to what extent NfL values varies during the disease course [55–61]. However, the increased availability of the latest ultrasensitive assay for the detection of NFs and the gathering of normative values, will likely allow an accurate distinction between these rarer, benign, forms of MND and ALS shortly.

**Table 1:** Key NfL studies for the diagnosis of MND. The table displays a list of studies dealing with NfL role in the differential diagnosis of MND according to current literature

Study	Biomatrix	Method	ALS sample size (n)	Disease controls (*n*)	Type of comparison	Sensitivity	Specificity	Cut-off value	AUC
Reijn, 2009 [65]	CSF	sandwich-ELISA	32	*ALS-mimic disorders (26)*	ALS vs ALS mimics	75%	79%	22.6 ng/L	0.79
Tortelli, 2012 [66]	CSF	ELISA test Uman Diagnostic AB; Umea, Sweden	37	*Neurodegenerative diseases (21)* CIDP (25)	ALS vs all non-ALS	78.4%	72.5%	1981 ng/l	0.79 (CI 0.69–0.87)
Steinacker, 2016 [64]	CSF	Elisa test IBL, HamburgGermany	253 (including 20 fALS and 11 PLS)	*MND mimics (85)* *Other neurological diseases (117)*	MND vs MND mimics	77% (CI 71–82%)	88% (CI 79% to 94%)	2200 pg/mL	0.866 ± 0.023 (CI 0.821–0.911)
					MND vs all non-MND	not reported	85% (CI 79 to 90%)	2200 pg/mL	0.851 ± 0.019 (CI 0.813 to 0.888)
Oeckl, 2016 [63]	CSF	Elisa test IBL, HamburgGermany	75 (5 for each center)	*Neurological controls from each center with variable diagnosis (76)*	cumulative dataset	79% (CI 66.1–88.6%)	86.4% (CI 75.7–93.6%)	1431 pg/mL	0.86 (CI 0.79–0.93)
					Paris measurement	81.1%	88.3%	2521 pg/mL	not reported
Poesen, 2017 [69]	CSF	ELISA test Uman Diagnostic AB; Umea, Sweden	220	*Disease controls (316)* including 10 normal controls *Disease mimics (50)*	ALS vs *Disease Controls* in training cohort	78.8% (71.4–85%)	72.7% (66–78.8%)	3819 pg/mL	0.809 (CI 0.763–0.849)
					ALS vs *Disease Controls* in validation cohort	88.4% (CI 78.8–94.0%)	84.7% (CI 76.8–90.2%)	3819 pg/mL	not reported
					ALS vs *Disease mimics*	85.4% (CI 78.8–90.6%)	78.0% (CI 64.0–88.5%)	2453 pg/mL	0.863 (CI 0.808–0.908)
Gaiani, 2017 [70]	CSF	ELISA test Uman Diagnostic AB; Umea, Sweden	94 ALS and 20 FTD	*Motor neuropathies (18) including CIDP (15) and MMN (3)*	ALS vs all other patients	81.9% (CI 74.5–89.4%)	80.5% (CI 71.9–89%)	1843.52 pg/mL	0.91 (CI 0.87–0.95)
				*Controls (44):* mononeuritis, primary headaches, and no objective signs of a neurologic disease	ALS vs controls	88.7% (CI 79.5–97.7%)	89.4% (CI 83–96%)	1380.48 pg/mL	0.96 (CI 0.92–0.99)
Feneberg 2018 [58]	CSF	Elisa test IBL, HamburgGermany	early phase (54)	*Other neurological diseases (65 CSF, 28 serum)* *MND mimics (27 CSF, 21 serum)* *Other MND (21 CSF, 16 serum)*	early symptomatic ALS vs other neurological diseases	94% (83–99%)	86% (75–93%)	2300 pg/mL	0.95 (0.91–0.99)
					early symptomatic ALS vs MND mimics	89% (71–98%)	94% (83–99%)	2183 pg/mL.	(0.94–1)
			late phase (135)		late symptomatic ALS vs other neurological diseases	89% (82–93%)	84% (73–92%)	2146 pg/mL	0.93 (0.9–0.96)
					late symptomatic ALS vs MND mimics	89% (71–98%)	89% (81–93%)	2089 pg/mL	0.96 (0.93–0.99)
	serum	SiMOA	early phase (45)		early symptomatic ALS vs other neurological diseases	88% (73–96%)	92% (80–94%)	128 pg/mL	0.92 (0.85–0.99)
					early symptomatic ALS vs MND mimics	100% (84–100%)	90% (76–97%)	97 pg/mL	0.99 (0.97–1)
			late phase (118)		late symptomatic ALS vs other neurological diseases	79% (CI 70–86%)	92% (80–98%)	116 pg /mL	0.9 (0.83–0.97)
					late symptomatic ALS vs MND mimics	100% (84–100%)	84% (76–90%)	95 pg/mL	0.97 (0.94–1)
Li, 2018 [60]	CSF	ELISA test Uman Diagnostic AB; Umea, Sweden	53 (35 early phase)	*Controls (32)ALS mimics (7) Other neurological diseases (25)*	ALS vs all non-ALS	96.2% (95% CI, 87–99.5)	56.3% (95% CI, 37.7–73.6)	1139 pg/mL	0.775 (CI, 0.671–0.858
					Early ALS vs all non-ALS	91.4% (CI 76.9–98.2)	59.4% (CI 40.6–76.3)	1307 pg/mL	0.772 (CI 0.654–0.866)
Rossi, 2018 [47]	CSF	ELISA test Uman Diagnostic AB; Umea, Sweden	190	*Control group 1 (82):* non-inflammatory, non-acute onset neurological disorders, including *ALS-mimic diseases* (31)	ALS vs *control group 1*	76.3% (CI 69.8–81.7)	72.8% (CI 69.2–80.9)	1838 ng/L	0.775 (CI 0.713–0.837)
					ALS vs *ALS mimics*	78.2% CI (71.2–83.5)	63.0% CI (44.1–78.4)	1540 ng/L	0.694 CI (0.572–0.817)
				*Control group 2 (48):* acute/subacute inflammatory disorders and tumors/metastases of the nervous system	ALS vs *control group 2*	79.2% CI (72.9–84.3)	41.3% CI (28.3–55.7)	1470 ng/L	0.542 CI (0.437–0.648)
Verde, 2019 [68]	serum	SiMOA	124	*Disease controls (44)* *Non-neurodegenerative controls (50)* *Neurodegenerative diseases (65)*	ALS vs disease controls	85.5% (CI 78–91.2%)	77.3% (CI 62.2 to 88.5%)	62 pg/mL	0.873 (CI 0.81 to 0.935
					ALS vs non neurodegenerative controls	89.5% (CI 82.7–94.3%)	92% (CI 80.8–97.8%)	49 pg/mL	0.971 (CI 0.95 to 0.991)
					ALS vs all non-ALS	85.5% (CI 78 to 91.2%)	81.8% (CI 74.9 to 87.4%)	62 pg/mL	0.887 (CI 0.849 to 0.926)
Gille, 2019 [71]	serum	ECL-based assay	149	PLS (11) PMA (6)	ALS vs PLS	80.5% (Ci 73.3–86.6)	90.9% (Ci 58.7–99.8)	88 pg/ml	0.89 (Ci 0.83–0.93)
					ALS vs PMA	81.2% (CI 74.0–87.1)	66.7% (CI 22.3–95.7)	86 pg/ml	0.71 (CI 0.63–0.78)
				*Disease controls (82):* GBS (48), CIDP (20) hSP (14)	ALS vs disease controls (hSP excluded)	not reported	63.2% (CI 50.7–74.6)	139 pg/ml	0.58 (CI 0.51–0.64)
					ALS vs hSP	89.3% (CI 83.1–93.7)	78.6% (CI 49.2–95.35)	55 pg/ml	0.84 (CI 0.78–0.90)
Kasai, 2019 [72]	plasma	SiMOA	discovery cohort: 29	*Non-neurological controls (29)* *NMD patients (46)*	ALS (discovery cohort) vs controls	not reported	not reported	not reported	0.6659
					ALS (validation cohort) vs NMD	not reported	not reported	not reported	0.7824
	CSF		validation cohort: 46		ALS (discovery cohort) vs controls	not reported	not reported	not reported	0.7206
					ALS (validation cohort) vs NMD	not reported	not reported	not reported	0.9012
Abu-Rumeileh, 2020 [67]	CSF	ELISA test IBL, HamburgGermany	80	*healthy controls (43)* *ALS mimics (46)*	ALS vs healthy controls	96.3%	97.7%	1207 pg/mL	0.981 ± 0.011
					ALS vs *ALS mimics*	91.7%	91.3%	1955 pg/ml	0.922 ± 0.031

*Abbreviations*: *ALS* amyotrophic Lateral Sclerosis, *AUC* area under the curve, *CI* confidential interval, *NfL* neurofilament light chain, *pNfH* neurofilament heavy chain, *CSF* cerebrospinal fluid, *ECL* elettrochemoluminescence assay, *ELISA* enzyme -linked immunoadsorbent assay, *MND* motor neuron disorder, *fALS* familiar ALS, *SiMoA* single-molecule array, *FTD* Fronto-Temporal-Dementia, *MMN* multifocal motor neuropathy, *PLS* primary lateral sclerosis, *CIDP* Chronic inflammatory demyelinating polyneuropathy, *hSP* hereditary spastic paraplegia, *GBS* Guillain-Barre Syndrome, *NMD* neuromuscular disease

Filling the gap of a general lack of data about NF release in body fluids in SMA, the introduction of gene therapies with antisense oligonucleotides delivered intrathecally has allowed the collection of many CSF and blood samples for NF quantification in these patients. In a large case series of infants affected by type 1 SMA, plasma pNfH levels were manifold higher than in healthy controls [76]. However, studies collecting NF levels in later-onset SMA type 2 and 3 revealed no substantial differences in NfL and pNfH concentrations between patients and healthy controls in both serum and CSF [77, 78].

**Fig. 2:**
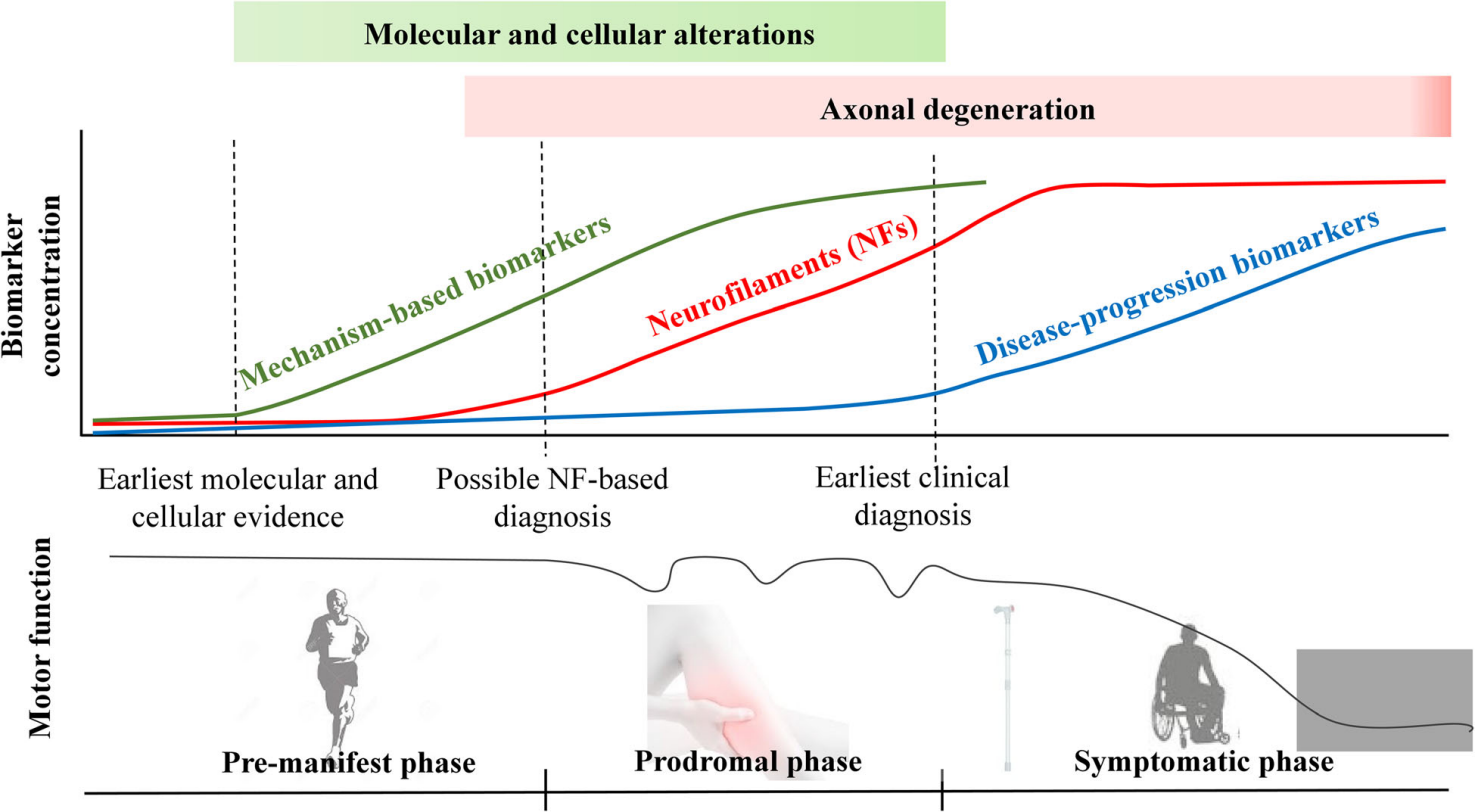
NFs and phases of the disease in ALS. Increased levels of NFs reflect axonal degeneration and can be detected in the prodromal phase of the disease, with the highest levels in the early symptomatic phase and seemingly stable levels as disease progresses. As the picture displays, during prodromal phase the neurodegenerative process has already begun but the patients generally complain of little disturbances (in the case of motor symptoms, for example, cramps or twitching) that do not compromise the overall function. Therefore, NFs analysis could have applications in the diagnosis, prognosis and early patient enrolment in clinical trials. However, we still need mechanism-based biomarkers that can be detected even earlier during the pre-manifest stage of the disease, when degeneration is not ongoing, and can inform us on targetable molecular and cellular alterations to be addressed in future clinical trials. An ideal disease-progression or pharmaco-dynamic biomarker (see text for explanation) would change with the progression of the disease. Doubts on the possible use of NFs as disease-progression biomarkers have been raised, but only few studies have thoroughly investigated their longitudinal behaviour

Importantly, the early increase of NFs in both serum and CSF allows a diagnosis of ALS at an early stage of the disease, i.e., within six months of symptom onset [58]. This addresses the core of the value of a diagnostic biomarker, namely the possibility to recognize the disease at a stage when interventions may still be disease-modifying. As suggested by Turner and Benatar [79] neurologists usually recognize this disease easily, also given the rarity of true mimics. Thus, a diagnostic biomarker would probably find its main application in guiding the general practitioner to seek an earlier referral to the MND specialist [79] or for patient recruitment into clinical trials at the earliest possible disease stage. Viewing ALS as a multi-step neurodegenerative disorder, with pre-clinical and prodromal phases during which neurodegeneration is already in place and progresses, NFs are candidate molecules mirroring this process revealing axonal damage before overt disease [59] (Fig. 2*)*.

Attempts to establish the pathobiology of NF release according to the involvement of upper and lower motor neuron showed a positive correlation between CSF pNfH and NfL levels and the overall number of regions affected by upper or lower MN signs [57, 67, 69] However, with respect of the relative contribution of UMN and LMN degeneration to the release of NF in body fluids, the studies conducted to date are not aligned. In one study, serum NfL correlated better with clinical signs of UMN than with those of LMN involvement [71]. Similarly, in another study, increasing levels of CSF NfL correlated with UMN burden and with corticospinal tract involvement, as depicted by lower fractional anisotropy and increased radial diffusivity by diffusion tract imaging [80]. However, subsequent larger studies provided controversial results on this issue, and, overall, did not confirm the correlation between NFs and DTI findings of corticospinal tract integrity [64, 70, 81]. Finally, in a recent study [67], CSF NfL concentration significantly correlated with the extent of LMN degeneration and not to that of UMN involvement, suggesting that the damaged motor neurons in the anterior horn of the spinal cord, which is rich in large axons, also significantly contribute to the release of NfL in CSF.

A possible explanation for the above-mentioned conflicting results in the current literature may be related to the inter-rater variability in the clinical evaluation of UMN or LMN signs. In our opinion, while attempts to clarify the mechanism of NF spill over by motor neurons should be encouraged since they might contribute to the understanding of the whole neurodegenerative process, it might be inappropriate to correlate NF levels to upper or lower MN involvement as defined exclusively by clinical signs. More objective neurophysiological and imaging techniques should be used to quantify the upper or lower motor neuron burden, though most of these measures will require within-centre standardization and validation.

### NFs as prognostic biomarkers in ALS and other MNDs

The prognostic value of NFs for ALS became evident since the first studies, given the positive correlation between NfL levels in CSF and some well-established clinical prognostic factors such as disease progression rate, clinical subtypes of MND and disease duration [56, 66, 70, 82]. This parallelism was later strengthened by other studies, holding for both NfL and NfH, in CSF and blood [57, 58, 67–69]. On the other hand, other known prognostic indicators as bulbar-onset, and cognitive involvement did not correlate as expected with NF levels [58, 70], possibly because the effect of neurodegenerative changes on NF levels is relatively independent of the brain region involved. Moreover, when patients were stratified in fast, intermediate and slow progressors by tertiles, according to the ALSFRS-r progression rate, baseline levels of NFs variably discriminated between these categories between studies [58, 67, 69, 83].

Although there is an overall, albeit not full, agreement about NfL levels being relatively stable over time since disease onset (Table 2), broader prospective and multi-centric studies will be needed to define more precisely the time course of NF release in relation with clinical staging. In our opinion, this added knowledge may help to clarify further the role of Nfs as markers of ongoing axonal degeneration in MNDs and, consequently, the time window for disease-modifying interventions. At the moment, however, the apparent lack of change of NFs concentration with advancing stages of diseases would indicate that the first measured value of NF would be informative about the rate of disease progression along the entire course of MND. The observation that baseline NF levels, i.e., at the first diagnostic visit, mostly in CSF [67, 70, 84], but also in serum [71, 83, 90] inversely correlated with survival in regression models support this conclusion. In univariate regression models, NF levels retain a high hazard risk for death [57, 70, 81]. More importantly, in multivariate models encompassing all other known prognostic factors such as ALSFRS-r, site of onset, age at onset, weight loss, and respiratory measures, only serum NfL concentration and some clinical variable different from one study to another (FVC [90] age, baseline ALSFRS-R, ΔFRS [86]), resulted as independent predictors of survival [90, 91]. Considering ALSFRS-R slope as the outcome, and taking into account baseline serum NfL and pNfH together with potential clinical predictors of prognosis (age, sex, C9ORF72 status, site of disease onset, baseline ALSFRS-R and ΔFRS), only progression rate resulted a meaningful clinical predictor. Nevertheless adding to the model baseline serum NfL, but not pNfH adds prognostic value not explainable otherwise (for every 1-point increase in log serum NfL level, the progression rate [92] is worsened by an additional 0.42 points/month [86]). Other studies supported the use of baseline serum or CSF NfL levels as an independent prognostic factor, with equal, if not superior value, of progression rate at diagnosis (delta-FS) [71], one of the most widely accepted clinical prognostic index computed at diagnosis [83]. A recent study showed significantly higher serum pNfH concentration in pyramidal, bulbar, and classic phenotypes (with the lowest concentrations in flail arm ALS, PMA, and PLS) and in more advanced cases (King’s stages 3 and 4 compared to King’s 1 and 2), with a positive correlation between pNfH and progression rate suggesting that a faster degeneration of the motor system is one of the determinants of serum pNfH concentration [91].

**Table 2:** Data on longitudinal behaviour of NFs in MND according to current literature. The table displays a list of studies with at least one longitudinal follow-up after the first observation for the neurofilament of interest; the main findings are highlighted in bold characters. Methods for NF quantification and the potential limits of these findings are briefly reported

Neurofilament isoform	Study	Biomatrix	Methods	Concentration range of the kit	Concentration range of cross-sectional results	Longitudinal sample size (n)	Genetic carriers (n)	Longitudinal data	Potential limits
NfL	Lu, 2015 [56]	serum, plasma and CSF	ECL-based assay	Linearity tested 1–50,000 pg/mL	IQR plasma: 54.4–158.4 pg/mL IQR serum: 54.5–151 pg/mL IQR CSF: 4376–11,736 pg/mL	Plasma: 67 Serum: 43 CSF: 24	excluded	plasma NfL does not change over time serum NfL have small ns increase over time: **stable NfL levels in blood for 15 mts follow-up** CSF NfL: small increase in fast and slow progressors	1) 3 cohorts testing different biomatrices, not related blood and CSF in the same patient. 2) Small sample for CSF collected longitudinally
	Skillback, 2017 [84]	CSF	In-house method [I method] and ELISA kit Uman Diagnostics [II method] (NF-light ELISA kit, UmanDiagnostics AB, Umea°, Sweden); normalization of the two methods	I method: detection limit 250 ng/mL II method: detection limit 125 ng/mL (linearity 125–16,000 ng/mL) III method: detection limit 50 ng/mL; linear correlation with previous methods but resulted in higher levels of NfL (normalization required)	970–3600 ng/mL	69	unknown	67% of MND showed **higher CSF NFL concentration at a later stage of disease**, those without rising levels had higher NFL at baseline	1) use of two different detection methods, the first less sensitive; 2) no notion of the timeline of longitudinal sampling (after how many months the second sample, in which phase of disease, etc)
	Poesen, 2017 [69]	CSF	pNfH: Biovendor, Brno, Czech Republic; NfL: UmanDiagnostics AB, Umea, Sweden; UD51001	pNFH: 62.5–4000 pg/ml NfL: 1–10,000 pg/mL	pNFH: 114–18,089 pg/mL NfL: 370–108,909 pg/mL	17	26 /220 in cross-sectional cohort not specified in longitudinal cohort	CSF **pNfH levels rather stable** over time, CSF **NfL increased** over time for a subset of **intermediate and fast disease progressors**	1) small sample for longitudinal observation; 2) sampling at different disease duration
	Benatar, 2018 [85]	serum	ECL-based assay	reference to previous work [50] 15.6–10,000 pg/mL	21–555 pg/mL	11	3	**stable levels of NfL** in sporadic ALS	1) small sample size; 2) follow-up very sparse without knowledge of clinical progression
	Verde, 2019 [68]	serum	SiMOA, time-normalized follow-up NfL levels	0.686–500 pg /mL [Quanterix]	14.6–908 pg / mL	29	8 / 224 in cross-sectional cohort	**Overall stable**: the variations in NfL from first to second sample ranged from −69.3 to + 189 pg/mL (median, −0.1 pg/mL).	small sample size
	Gille, 2019 [71]	serum	ECL-based assay	reference to previous work [50] 15.6–10,000 pg/ml	0.3–1141 pg/mL	16	15 / 149 in cross-sectional cohort	**relatively stable over time**, although an increase could be observed within the first 20 months after onset of disease, irrespective of the disease progression rate	1) small sample for longitudinal observation; 2) sampling at different disease duration; 3) no information of pNfH levels related to clinical outcome
	Benatar, 2020 [86]	serum	SiMOA	0.686–500 pg /mL [Quanterix]	2–369 pg/mL	106	8	relatively stable over time, computation of longitudinal trajectories by average slope: 0.011 log units/ month (95% CI, −0.054 to 0.076)	well-conducted prospective longitudinal study representative of ALS population; the only potential limit might be sample collection was at some latency from diagnosis (average: 0.7 year), and the study population was skewed towards a slow progression (ALSFRS-r slope: 0.65/month) than general ALS population (1/month, PRO-ACT)
pNfH	Lu, 2015 [82]	plasma	in-house ELISA for hyperphosphorylated NfH (NfHSMI34) and variably phosphorylated NfH (NfHSMI35)	linearity tested for serial dilutions with urea pre-analytical tretament to overcome the “hook effect”, tested in normalized optical density (NOD)	not reported	74	7 / 136 in cross-sectional cohort	**slight increase** in plasma pNfH for **slow progressors** (32), **no change** for **intermediate** progressors (24), **slight decrease** for **fast progressors** (32)	in-house immunoassay, now better technologies for pNfH quantification
	McCombe, 2015 [87]	serum	ELISA kit EnCor Biotechnology Inc., Gainesville, FL, USA	linearity not tested; from the manufacturer, linearity for standard curve: 0.156–10 ng/mL	Not reported	98	not reported	**overall rise and then fall** in pNfH levels for ALS pts. with at least 3 longitudinal samples; different trajectories according to survival	potentially better immunodetection techniques
	Gendron, 2017 [88]	CSF	Meso Scale Discovery immunoassay with mouse antihuman pNFH anti-body and a sulfo-tagged polyclonal anti-pNFH antibody as capture and detection antibodies	not reported	50–3119 pg/mL non c9-ALS or non c9-ALS-FTD 117–4671 pg/mL for c9-ALS or c9-ALS-FTD	44	27	**no change** in longitudinally collected pNfH in c9ALS, c9ALS-FTD (both groups: 27 pts) and non-c9 ALS (17 pts); no difference between fast and slow progressors	1) small sample size; 2) no information of pNfH levels related to clinical outcome
	Benatar, 2019 [89]	serum	CE marked ELISA (Euroimmun AG, Lubeck, Germany)	9.4–1000 pg/mL	639–11,418 pg/mL	16	10	**stable levels** of serum NfH in ALS pts.; among mutation carriers who convert there is an increase in serum pNfH in advance of the appearance of manifest disease	1) small sample for longitudinal observation; 2) sampling at different disease duration; 3) no information of pNfH levels related to clinical outcome
	Benatar, 2020 [86]	serum	SiMOA	0–2000 pg/mL	1–976 pg/mL	106	8	relatively stable over time, computation of longitudinal trajectories by average slope: 0.006 log units/month month (95% CI, −0.063 to 0.084)	well-conducted prospective longitudinal study representative of ALS population; the only potential limit might be sample collection was at some latency from diagnosis (average: 0.7 year), and the study population was skewed towards a slow progression (ALSFRS-r slope: 0.65/month) than general ALS population (1/month, PRO-ACT)

*Abbreviations*: *NfL* neurofilament light chain, *pNfH* neurofilament heavy chain, *CSF* cerebrospinal fluid, *ECL* elettrochemoluminescence assay, *ELISA* enzyme -linked immunoadsorbent assay, *ns* not significant, *MND* motor neuron disorder, *mts* months, *pts.* patients, *SiMoA* single-molecule array

This further sustains the potential role of NFs for stratification and the correct allocation of patients in clinical trials.

Regarding other MNDs, there is an overall lack of data because of the rarity of these conditions.

Cross-sectional levels of NFs are generally measured at different time points from the onset of disease, without full knowledge of their longitudinal behaviour. Of course, the diagnosis of each of these conditions has by itself robust prognostic implications, when compared to ALS [74].

### Neurofilaments in genetically confirmed ALS

NF levels did not differ between sporadic and familial forms of ALS when the genetic cases were analysed as a single homogeneous group, irrespective of the causing mutation [64, 83]. However, the comparison between large cohorts of c9orf72-expanded ALS and sporadic ALS patients, revealed significantly higher CSF levels of pNfH in the former group, correlating with shorter survival and a more severe disease course [88] . C9orf72 ALS has a faster disease progression rate and shorter survival, reflecting a widespread CNS neurodegeneration and more severe brain atrophy [91] that may explain higher pNfH concentration rather than a specific role of C9orf72. In this subgroup of patients, the search for the poly (GP), a dipeptide repeat protein resulting from repeat-associated non-ATG (RAN) translation of C9ORF72, provides the most specific marker for the hexanucleotide expansion, while NfL may be used in association, especially to reveal the onset of neuronal degeneration in pre-symptomatic carriers [93]. In this regard, the expanded knowledge of the genetic landscape of ALS has recently provided the opportunity to analyse potential biomarkers in large cohorts of pre-symptomatic carriers by longitudinal follow-up during prodromal stages. In the seminal work of Benatar et al. [85], pre-symptomatic carriers of known disease-causing mutations (e.g. SOD1, C9orf72, TARDBP, FUS, VCP) showed rising levels of serum NfL up to 11.6 months before “phenoconversion” (i.e., the onset of motor symptoms typical of ALS). Importantly, serum NfL levels continued to rise until six months after disease onset and then stabilized [85]. The increased sensitivity of the last-generation assays, and the prospective nature of the study allowed to establish that the concentration of serum NfL during prodromal stages in converters was well-above that of age-matched controls, which was in contrast to a previous study in which NF levels in carriers of ALS-causing mutations were not elevated till symptom onset [94]. In the same cohort of carriers, including four additional phenoconverters, the analysis of pNfH in serum and CSF allowed further correlations between genotype and the initiation of the neurodegenerative process [89] Serum NfL was more sensitive than pNfH in detecting ongoing neurodegeneration as its levels were higher than normative values in the majority of converters, while for pNfH this was true only for one patient. Among converters, it was shown that NF release could be dated back between 6 and 12 months in SOD1 mutation carriers, whereas in a single FUS c.521 del 6 converter and two C9ORF72 expansion carriers the same effect was observed respectively 2 and 3.5 years before phenoconversion [89]. Taken together these data may suggest that the duration of the pre-symptomatic phase may be directly correlated to the disease course (the longer the presymptomatic phase, the slower the disease progression), and may predict disease onset [89].

Overall, these findings shed light on the natural history of ALS neurodegenerative process driven by specific mutations and may guide the timing of future genetic therapies in mutation carriers, monitoring their effect.

### NFs as disease-progression biomarkers

While the search for disease-modifying therapies in ALS is ongoing, NFs have been increasingly taken into consideration as part of the outcome measures. Starting from gene therapies, the administration of antisense oligonucleotides (ASO) in mouse models of mutated SOD1, prolonged survival and ameliorated clinical conditions, in association with increased amplitude of compound muscle action potentials (cMAPs) as well as a reduction in the release of pNfH in serum [95]. On the same line, the use of NFs in CSF and serum as the primary outcome measure of experimental drugs testing has been introduced or planned in other clinical trials [96–100]; other trials identified by ClinicalTrials.gov Identifier: NCT02118727; NCT03800524; NCT02872142].

The advent of ASO therapy for SMA represented another example of how biomarker research can contribute to clinical studies. First published studies on NF levels in SMA type 1 patients revealed an initial decline of plasma pNfH after Nusinersen administration, followed by a plateau. Moreover, younger-onset infants had reduced cMAP at baseline, pointing to a correlation between pNfH and markers of disease severity [77] These results were confirmed in a small group of type 1 SMA infants where NfL measured in CSF (collected at the time of lumbar puncture for ASO administration), starting from baseline peak levels, normalized between the fourth and fifth dose of Nusinersen, and the decrease in its levels correlated with improvement in motor function [101]. However, studies in SMA types 2 and 3 showed that NF levels did not differ between patients and controls in both CSF and serum, and their concentrations do not substantially modify during intrathecal therapy with ASO [77, 78].

Based on the previous observations that NF levels in ALS remain stable during disease progression, reaching their maximal levels probably soon after disease onset (see Table 2), some doubted that NFs, would be able to show a biological response during clinical trials. This would be in contrast to other biomarkers such as, for example, of urinary p75^ECD^, whose levels rise during the course of disease [102]. Indeed, according to a strict definition, NFs can be considered neither pharmacodynamic biomarkers, (defined as biomarkers that reliably change in response to treatment as a consequence of the effect of an experimental drug on a pathological pathway), nor disease progression markers (intended as serial measures that change with the worsening of the disease) [110]. Nevertheless, in the abovementioned study on mutated SOD1 mouse models, serum pNfH was shown to increase in the placebo group, while mice treated with ASOs had stable levels of the marker [95].

Furthermore, in humans, a phase 1–2 ascending dose trial evaluating the effect of Tofersen (an ASO that mediates SOD1 degradation) in SOD1 ALS, exploratory results on 50 SOD1 mutated patients showed a slowing in the decrease of ALSFRS-R, slow vital capacity and handheld dynamometry scores during time, a matched decrease of SOD1 protein in the CSF and of pNfH and NfL in plasma and CSF, especially in the group treated with the highest dose (100 mg) and in fast progressors, from baseline to day 85 and to a lesser extent also later on [103]. Notably, in the placebo group plasma and CSF NFs levels remained almost stable.

However, a positive biological response, detectable at the level of a molecular or cellular biomarker, might not always correspond to clinical improvement. For example, trials in HIV and AD succeeded in reducing respectively retroviral load [96] and the amyloid-β plaque burden [104], but were clinically unsuccessful.

Current clinical outcome measures in use in clinical trials for ALS imply prolonged and expensive follow-up time before a conclusion can be drawn.

**Fig. 3:**
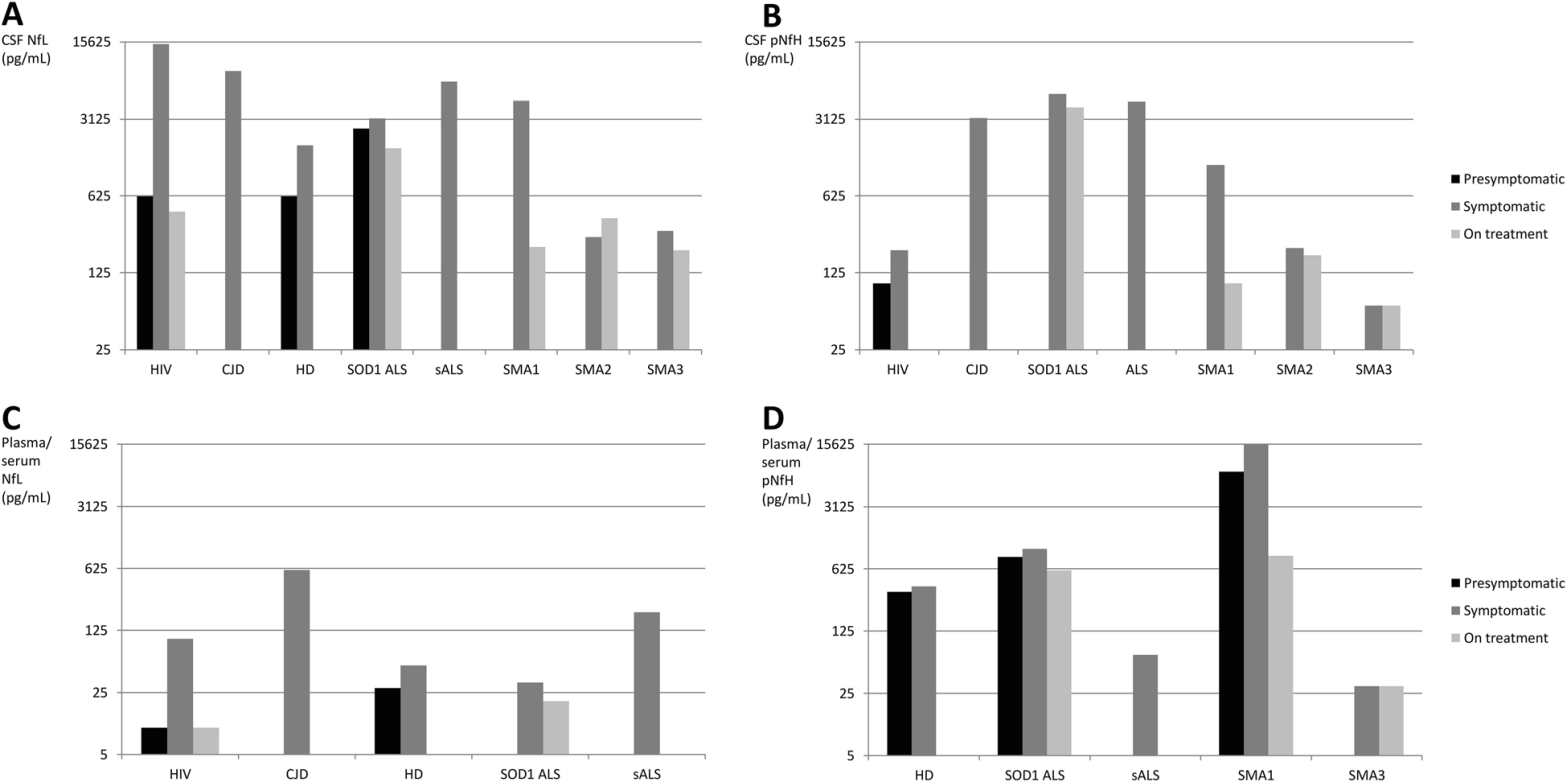
The increase of cerebrospinal fluid and plasma/serum neurofilament light and heavy chain in different phases of a variety of neurodegenerative diseases. The figure shows the increase of cerebrospinal fluid and plasma/serum neurofilament light (NfL) and heavy (NfH) chain in different phases (presymptomatic, symptomatic and after treatment) of a variety of neurodegenerative diseases associated with axonal damage. Columns represent mean values reported before symptoms onset (black), after symptom onset (grey), and after treatment (light grey). As far as HIV is concerned, the onset refers to neurological impairment, and the presymptomatic phase refers to neuro-asymptomatic status. As far as treatment is concerned, it refers to Nusinersen treatment for SMA, Tofersen treatment for SOD1 ALS and ART for HIV. The specific reference list is reported in online supplementary material. Abbreviations: ALS, amyotrophic lateral sclerosis; CJD, Creutzfeldt-Jakob disease; CSF, cerebrospinal fluid; HD, Huntington’s Disease; NfL, neurofilament light chain; pNfH, neurofilament heavy chain, SMA, spinal muscular atrophy

Using a simulation study Benatar et al. [86] found that the sample size for an ALS trial would be reduced by 8.2% if adding baseline serum NfL measurements as covariates, whereas using serum NfL as a pharmacodynamic biomarker may allow significant sample size reduction compared to more traditional phase 2 studies in which changes in the ALSFRS-R are used as the primary endpoint. There is a lack of similar data on CSF NFs. Figure 3 shows NFs behavior in different phases of a variety of neurodegenerative diseases.

More extensive multicentric studies on the longitudinal behaviour of NF based on standardized techniques are warranted to assess further the role of NFs as disease progression biomarkers. Furthermore, the actual potential of NFs as pharmacodynamic biomarkers will become apparent once effective treatments are available for ALS.

### Limitations and future directions

Despite the technological advancement and the spreading of the use of NF as a biomarker of MNDs, most of the metanalyses and transversal studies on NF indicate that these biomarkers merely reflect the degree of axonal loss, and are non-disease specific [23, 24, 105]. Nevertheless, NF levels are several folds higher in ALS compared to other neurodegenerative diseases, with the sole exception of HIV-associated dementia and Creutzelfeldt-Jacob disease [23, 54, 106, 107].

Ideally, to shorten the diagnostic latency for ALS and other MNDs, the biomarker should rapidly address the patient from the general practitioner to the neurologist, to accelerate the diagnosis (e.g. for ALS at an early stage when El Escorial revised criteria may not be completely fulfilled). However, the application of NF quantification at the general practitioner or first aid room may confuse rather than expedite the diagnosis of MND. On the other hand, a recent study proved that serum pNfH was already elevated above normative values in individuals admitted with early motor symptoms who only later satisfied the diagnosis of sporadic or familial ALS [108] . The lack of specificity of NFs towards neurodegenerative conditions should not alarm against their diagnostic value as far as their use is limited to experienced neurologists who can orient the diagnosis in the right direction. With these premises, NFs measurement in a specialized neurological setting can be helpful for early trial enrolment, catching the disease in the first phases, when potentially drugs or interventions may have the greatest effect.

Fourth-generation immunoassays have enhanced our ability to detect low concentrations of NFs in peripheral biofluids. This is especially true for NfL, which is hardly detectable in blood samples using standard ELISA assays. However, these technologies are sophisticated and expensive, and not easily accessible to most clinical centres. Thus, more affordable immunoassays should be developed to allow NF analysis becoming routine in clinical practise.

The stability of NF levels along the course of ALS might represent a limitation of these biomarkers as it may shadow the recognition of the beneficial effect of therapeutics. However, the longitudinal behaviour of NFs has been investigated to date only in a few studies without standardized techniques or protocols and requires validation with confirmatory studies. Additionally, different NF trajectories might occur according to the disease progression rate [82], suggesting that the pace of neuroaxonal damage varies according to the extent of neurodegeneration. Finally, it has been speculated that the occurrence of adaptive phenomena as transcriptional or translational modifications of NF subunits may imply a compensatory over-expression of NfL over the other isoforms within the degenerating motor neuron in order to save energy [40].

Despite the expanding literature on the role as biomarkers in ALS and other neurodegenerative disorders, several issues remain to be addressed. In the first place, with a few exceptions, most studies are single-centre [58, 69, 88] and used various immunodetection techniques, which often resulted in contrasting results. For example, the initial finding of normal NF levels in pre-symptomatic c9orf72 patients [88] was later disproved [85]. The already mentioned variability in NfL and NfH levels during the disease course clearly indicates that additional work is needed to clarify the pace of change of NF and its correlations with clinical measures.

As Turner and Benatar pointed out in their position paper in 2015 [79], a concept reiterated in the White Paper of the Society of CSF Analysis and Clinical Neurochemistry [109], the biomarker development process must step forward from the discovery platform aiming to clinical validation and then clinical implementation [109]. These themes must be addressed by international cooperative efforts and multi-players exchange platforms, where academia and clinicians can dialogue on how to efficiently employ the most promising biomarkers. Finally, findings need to be replicated until global laboratory standardization as well as a clinical meaningful use have been established for a putative molecule.

## Conclusions

In conclusion, NFs represent very promising biomarkers for MNDs, which help in the differentiation between ALS and more prognostically favorable MND, and allow the diagnosis of ALS when clinical criteria are not yet fulfilled. On a large scale, clinical research will be implemented thanks to early recruitment and proper stratification in subgroups with different prognosis. In the future, when targeted genetic therapies will be available, serial monitoring of NFs may identify the beginning of the neurodegenerative process in carriers of known disease-causing mutations, guiding the decision to start such therapies. The advent of disease-modifying therapies for SMA, as Nusinersen, has allowed the demonstration that in SMA type 1, NFs concentration decreases proportionally to the cMAP increase, whereas in type 2 and 3 NFs are not elevated, and Nusinersen administration does not substantially modify their levels.

It might thus be inferred that NF levels will be useful to monitor disease progression only if their levels are raised from the start. Nevertheless, these speculations need to be substantiated by more extensive multicentric studies, with systematic longitudinal follow-up and robust clinical correlations. In our opinion, this might be warranted by parallel clinical and observational research, aiming to contextualize the NF role during the various phases of clinical management (i.e., diagnosis, follow-up, treatment).

At the same time, the search for alternative biomarkers should continue. NF release into biofluids is the final event in the process that leads to the degeneration of motor neurons. We need mechanism-based biomarkers that we can detect even earlier than the prodromal phase of the disease and can inform us on molecular and cellular alterations, providing new therapeutic targets to be addressed in future clinical trials (Fig. 2).

## Supplementary information

**Additional file 1.**

## Data Availability

Not applicable.

## Abbreviations

AD: Alzheimer’s disease
ALS: Amyotrophic lateral sclerosis
ASO: Antisense oligonucleotides
CSF: Cerebrospinal fluid
ECL: Electrochemiluminescence
ELISA: Enzyme-linked immunosorbent assay
hSP: Hereditary spastic paraparesis
LMN: Lower motor neuron
MND: Motor neuron disorder
NF: Neurofilaments (all the isoforms)
NfL: Neurofilament light chain
NfM: Neurofilament medium chain
NfH: Neurofilament heavy chain
PLS: Primary lateral sclerosis
PMA: Progressive muscular atrophy
SBMA: Spino-bulbar muscular atrophy
SDS: Sodium dodecyl sulphate
SiMoA: Single-molecule array
SMA: Spinal muscular atrophy
UMN: Upper motor neuron
