# Supplementary material for: Neurofilaments in motor neuron disorders: towards promising diagnostic and prognostic biomarkers
Source: Mol Neurodegener. 2020 Oct 15;15:58. doi: 10.1186/s13024-020-00406-3 (PMC7559190; doi:10.1186/s13024-020-00406-3)

**References list for figure 3**

1. Feneberg E, Oeckl P, Steinacker P, Verde F, Barro C, Van Damme P, et al. Multicenter evaluation of neurofilaments in early symptom onset amyotrophic lateral sclerosis. Neurology. 2018;90:e22-e30. doi: 10.1212/WNL.0000000000004761.
2. Darras BT, Crawford TO, Finkel RS, Mercuri E, De Vivo DC, Oskoui M, et al. Neurofilament as a potential biomarker for spinal muscular atrophy. Ann Clin Transl Neurol. 2019;6:932-944. doi: 10.1002/acn3.779.
3. Wurster CD, Günther R, Steinacker P, Dreyhaupt J, Wollinsky K, Uzelac Z, et al. Neurochemical markers in CSF of adolescent and adult SMA patients undergoing nusinersen treatment. Ther Adv Neurol Disord 2019; 12:1756286419846058. doi: 10.1177/1756286419846058.
4. Wurster CD, Steinacker P, Günther R, Koch JC, Lingor P, Uzelac Z, et al. Neurofilament light chain in serum of adolescent and adult SMA patients under treatment with nusinersen. J Neurol. 2020;267:36-44. doi: 10.1007/s00415-019-09547-y.
5. Benatar M, Zhang L, Wang L, Granit V, Statland J, Barohn R, et al. Validation of serum neurofilaments as prognostic and potential pharmacodynamic biomarkers for ALS. Neurology. 2020;95:e59-e69. doi:10.1212/WNL.0000000000009559
6. Benatar M, Wuu J, Andersen PM, Lombardi V, Malaspina A. Neurofilament light: A candidate biomarker of presymptomatic amyotrophic lateral sclerosis and phenoconversion. Ann Neurol. 2018;84:130-139. doi: 10.1002/ana.25276.
7. Benatar M, Wuu J, Lombardi V, Jeromin A, Bowser R, Andersen PM, Malaspina A. Neurofilaments in pre-symptomatic ALS and the impact of genotype. Amyotroph Lateral Scler Frontotemporal Degener. 2019;20:538-548. doi: 10.1080/21678421.2019.1646769.
8. Olsson B, Alberg L, Cullen NC, Michael E, Wahlgren L, Kroksmark AK, et al. NFL is a marker of treatment response in children with SMA treated with nusinersen. J Neurol. 2019;266:2129-2136. doi: 10.1007/s00415-019-09389-8.
9. Miller T, Cudkowicz M, Shaw PJ, Andersen PM, Atassi N, Bucelli RC, et al. Phase 1-2 Trial of Antisense Oligonucleotide Tofersen for SOD1 ALS. N Engl J Med. 2020;383:109-119. doi:10.1056/NEJMoa2003715
10. Abu-Rumeileh S, Capellari S, Stanzani-Maserati M, Polischi B, Martinelli P, Caroppo P, et al. The CSF neurofilament light signature in rapidly progressive neurodegenerative dementias. Alzheimers Res Ther. 2018;10:3. doi:10.1186/s13195-017-0331-1
11. De Vivo DC, Bertini E, Swoboda KJ, Hwu WL, Crawford TO, Finkel RS, et al. Nusinersen initiated in infants during the presymptomatic stage of spinal muscular atrophy: Interim efficacy and safety results from the Phase 2 NURTURE study. Neuromuscul Disord. 2019;29:842-856. doi: 10.1016/j.nmd.2019.09.007.
12. Totzeck A, Stolte B, Kizina K, Bolz S, Schlag M, Thimm A, et al. Neurofilament Heavy Chain and Tau Protein Are Not Elevated in Cerebrospinal Fluid of Adult Patients with Spinal Muscular Atrophy during Loading with Nusinersen. Int J Mol Sci. 2019;20:5397. doi: 10.3390/ijms20215397.
13. Faravelli I, Meneri M, Saccomanno D, Velardo D, Abati E, Gagliardi D, et al. Nusinersen treatment and cerebrospinal fluid neurofilaments: An explorative study on Spinal Muscular Atrophy type 3 patients. J Cell Mol Med. 2020;24:3034-3039. doi: 10.1111/jcmm.14939.
14. Walter MC, Wenninger S, Thiele S, Stauber J, Hiebeler M, Greckl E, et al. Safety and Treatment Effects of Nusinersen in Longstanding Adult 5q-SMA Type 3 - A Prospective Observational Study. J Neuromuscul Dis. 2019;6:453-465. doi: 10.3233/JND-190416.
15. Hermansson L, Yilmaz A, Axelsson M, Blennow K, Fuchs D, Hagberg L, et al. Cerebrospinal fluid levels of glial marker YKL-40 strongly associated with axonal injury in HIV infection. J Neuroinflammation. 2019;16:16. doi: 10.1186/s12974-019-1404-9.
16. Wild EJ, Petzold A, Keir G, Tabrizi SJ. Plasma neurofilament heavy chain levels in Huntington's disease. Neurosci Lett. 2007 May 7;417:231-3. doi: 10.1016/j.neulet.2007.02.053.
17. Anderson AM, Easley KA, Kasher N, Franklin D, Heaton RK, Zetterberg H, et al. Neurofilament light chain in blood is negatively associated with neuropsychological performance in HIV-infected adults and declines with initiation of antiretroviral therapy. J Neurovirol. 2018;24:695-701. doi: 10.1007/s13365-018-0664-y.
18. Anesten B, Yilmaz A, Hagberg L, Zetterberg H, Nilsson S, Brew BJ, et al. Blood-brain barrier integrity, intrathecal immunoactivation, and neuronal injury in HIV. Neurol Neuroimmunol Neuroinflamm. 2016;3:e300. doi: 10.1212/NXI.0000000000000300.
19. Ulfhammer G, Edén A, Mellgren Å, Fuchs D, Zetterberg H, Hagberg L, et al. Persistent central nervous system immune activation following more than 10 years of effective HIV antiretroviral treatment. AIDS. 2018;32:2171-2178. doi: 10.1097/QAD.0000000000001950.
20. Gisslén M, Price RW, Andreasson U, Norgren N, Nilsson S, Hagberg L, et al. Plasma Concentration of the Neurofilament Light Protein (NFL) is a Biomarker of CNS Injury in HIV Infection: A Cross-Sectional Study. EBioMedicine. 2015;3:135-140. doi: 10.1016/j.ebiom.2015.11.036.
21. McGuire JL, Gill AJ, Douglas SD, Kolson DL; CNS HIV Anti-Retroviral Therapy Effects Research (CHARTER) group. Central and peripheral markers of neurodegeneration and monocyte activation in HIV-associated neurocognitive disorders. J Neurovirol. 2015;21:439-48. doi: 10.1007/s13365-015-0333-3.
22. Scahill RI, Zeun P, Osborne-Crowley K, Johnson EB, Gregory S, Parker C, et al. Biological and clinical characteristics of gene carriers far from predicted onset in the Huntington's disease Young Adult Study (HD-YAS): a cross-sectional analysis. Lancet Neurol. 2020;19:502-512. doi: 10.1016/S1474-4422(20)30143-5.
23. Byrne LM, Rodrigues FB, Johnson EB, Wijeratne PA, De Vita E, Alexander DC, et al. Evaluation of mutant huntingtin and neurofilament proteins as potential markers in Huntington's disease. Sci Transl Med. 2018;10:eaat7108. doi: 10.1126/scitranslmed.aat7108.
24. Byrne LM, Rodrigues FB, Blennow K, Durr A, Leavitt BR, Roos RAC, et al. Neurofilament light protein in blood as a potential biomarker of neurodegeneration in Huntington's disease: a retrospective cohort analysis. Lancet Neurol. 2017;16:601-609. doi: 10.1016/S1474-4422(17)30124-2.
25. Steinacker P, Blennow K, Halbgebauer S, Shi S, Ruf V, Oeckl P, et al. Neurofilaments in blood and CSF for diagnosis and prediction of onset in Creutzfeldt-Jakob disease. Sci Rep. 2016;6:38737. doi: 10.1038/srep38737.
26. Thompson AGB, Luk C, Heslegrave AJ, Zetterberg H, Mead SH, Collinge J, Jackson GS. Neurofilament light chain and tau concentrations are markedly increased in the serum of patients with sporadic Creutzfeldt-Jakob disease, and tau correlates with rate of disease progression. J Neurol Neurosurg Psychiatry. 2018;89:955-961. doi: 10.1136/jnnp-2017-317793.

**Table 1. Key NfL studies for the diagnosis of MND.** The table displays a list of studies dealing with NfL role in the differential diagnosis of MND according to current literature.

| Study | Biomatrix | Method | ALS sample size (n) | Disease controls (n) | Type of comparison | Sensitivity | Specificity | Cut-off value | AUC |
| --- | --- | --- | --- | --- | --- | --- | --- | --- | --- |
| Reijn, 2009  [61] | CSF | sandwich-ELISA | 32 | *ALS-mimic disorders (26)* | ALS vs ALS mimics | 75% | 79% | 22.6 ng/L | 0.79 |
| Tortelli, 2012  [62] | CSF | ELISA test Uman Diagnostic AB; Umea, Sweden | 37 |  | ALS vs all non-ALS | 78.4% | 72.5% | 1981 ng/l | 0.79 (CI 0.69–0.87) |
|  |  |  |  | *Neurodegenerative diseases (21)*  CIDP (25) |  |  |  |  |  |
| Steinacker, 2016  [60] | CSF | Elisa test IBL, HamburgGermany | 253  (including 20 fALS and 11 PLS) | *MND mimics (85)*  *Other neurological diseases (117)* | MND vs MND mimics | 77%  (CI 71-82%) | 88% (CI 79% to 94%) | 2200 pg/mL | 0.866±0.023 (CI 0.821 - 0.911) |
|  |  |  |  |  | MND vs all non-MND | not reported | 85% (CI 79% to 90%) | 2,200 pg/mL | 0.851±0.019 (CI 0.813 to 0.888) |
| Oeckl, 2016  [59] | CSF | Elisa test IBL, HamburgGermany | 75 (5 for each center) | *Neurological controls from each center with variable diagnosis (76)* | cumulative dataset | 79% (CI 66.1-88.6%) | 86.4% (CI 75.7% - 93.6%) | 1,431 pg/mL | 0.86 (CI 0.79 - 0.93) |
|  |  |  |  |  | Paris measurement | 81.1% | 88.3% | 2,521 pg/mL | not reported |
| Poesen, 2017  [65] | CSF | ELISA test Uman Diagnostic AB; Umea, Sweden | 220 | *Disease controls (316)* including 10 normal controls  *Disease mimics (50)* | ALS vs *Disease Controls* in training cohort | 78.8% (71.4-85%) | 72.7% (66-78.8%) | 3,819 pg/mL | 0.809 (CI 0.763-0.849) |
|  |  |  |  |  | ALS vs *Disease Controls* in validation cohort | 88.4% (CI 78.8%–94.0%) | 84.7% (CI 76.8%–90.2%) | 3,819 pg/mL | not reported |
|  |  |  |  |  | ALS vs *Disease mimics* | 85.4% (CI 78.8%–90.6%) | 78.0% (CI 64.0%–88.5%) | 2,453 pg/mL | 0.863 (CI 0.808-0.908) |
| Gaiani, 2017  [75] | CSF | ELISA test Uman Diagnostic AB; Umea, Sweden | 94 ALS  and 20 FTD | *Motor neuropathies (18) including CIDP (15) and MMN (3)* | ALS vs all other patients | 81.9% (CI 74.5%-89.4%) | 80.5% (CI 71.9%-89%) | 1,843.52 pg/mL | 0.91 (CI 0.87-0.95) |
|  |  |  |  | *Controls (44):* mononeuritis, primary headaches, and no objective signs of a neurologic disease | ALS vs controls | 88.7% (CI 79.5%-97.7%) | 89.4% (CI 83%-96%) | 1,380.48 pg/mL | 0.96 (CI 0.92-0.99) |
| Feneberg 2018  [54] | CSF | Elisa test IBL, HamburgGermany | early phase (54) | *Other neurological diseases (65 CSF, 28 serum)*  *MND mimics (27 CSF, 21 serum)*  *Other MND (21 CSF, 16 serum)* | early symptomatic ALS vs other neurological diseases | 94% (83%–99%) | 86% (75%–93%) | 2,300 pg/mL | 0.95 (0.91–0.99) |
|  |  |  |  |  | early symptomatic ALS vs MND mimics | 89% (71%–98%) | 94% (83%–99%) | 2,183 pg/mL. | (0.94–1) |
|  |  |  | late phase (135) |  | late symptomatic ALS vs other neurological diseases | 89% (82%–93%) | 84% (73%–92%) | 2,146 pg/mL | 0.93 (0.9–0.96) |
|  |  |  |  |  | late symptomatic ALS vs MND mimics | 89% (71%–98%) | 89% (81%–93%) | 2,089 pg/mL | 0.96 (0.93–0.99) |
|  | serum | SiMOA | early phase (45) |  | early symptomatic ALS vs other neurological diseases | 88% (73%–96%) | 92% (80%–94%) | 128 pg/mL | 0.92 (0.85–0.99) |
|  |  |  |  |  | early symptomatic ALS vs MND mimics | 100% (84%–100%) | 90% (76%–97%) | 97 pg/mL | 0.99 (0.97–1) |
|  |  |  | late phase (118) |  | late symptomatic ALS vs other neurological diseases | 79% (CI 70%–86%) | 92% (80%–98%) | 116 pg /mL | 0.9 (0.83–0.97) |
|  |  |  |  |  | late symptomatic ALS vs MND mimics | 100% (84%–100%) | 84% (76%–90%) | 95 pg/mL | 0.97 (0.94–1) |
| Li, 2018  [56] | CSF | ELISA test Uman Diagnostic AB; Umea, Sweden | 53  (35 early phase) | *Controls (32)*  *ALS mimics (7)*  *Other neurological diseases (25)* | ALS vs all non-ALS | 96.2% (95% CI, 87–99.5) | 56.3% (95% CI, 37.7–73.6) | 1,139 pg/mL | 0.775 (CI, 0.671–0.858 |
|  |  |  |  |  | Early ALS vs all non-ALS | 91.4% (CI 76.9–98.2) | 59.4% (CI 40.6–76.3) | 1,307 pg/mL | 0.772 (CI 0.654–0.866) |
| Rossi, 2018  [43] | CSF | ELISA test Uman Diagnostic AB; Umea, Sweden | 190 | *Control group 1 (82):* non-inflammatory, non-acute onset neurological disorders, including *ALS-mimic diseases* (31) | ALS vs *control group 1* | 76.3% (CI 69.8–81.7) | 72.8% (CI 69.2–80.9) | 1,838 ng/L | 0.775 (CI 0.713–0.837) |
|  |  |  |  |  | ALS vs *ALS mimics* | 78.2% CI (71.2–83.5) | 63.0% CI (44.1–78.4) | 1,540 ng/L | 0.694 CI (0.572–0.817) |
|  |  |  |  | *Control group 2 (48):* acute/subacute inflammatory disorders and tumors/metastases of the nervous system | ALS vs *control group 2* | 79.2% CI (72.9–84.3) | 41.3% CI (28.3–55.7) | 1,470 ng/L | 0.542 CI (0.437–0.648) |
| Verde, 2019  [64] | serum | SiMOA | 124 | *Disease controls (44)*  *Non-neurodegenerative controls (50)*  *Neurodegenerative diseases (65)* | ALS vs disease controls | 85.5% (CI 78-91.2%) | 77.3% (CI 62.2% to 88.5%) | 62 pg/mL | 0.873 (CI 0.81 to 0.935 |
|  |  |  |  |  | ALS vs non neurodegenerative controls | 89.5% (CI 82.7- 94.3%) | 92% (CI 80.8-97.8%) | 49 pg/mL | 0.971 (CI 0.95 to 0.991) |
|  |  |  |  |  | ALS vs all non-ALS | 85.5% (CI 78% to 91.2%) | 81.8% (CI 74.9% to 87.4%) | 62 pg/mL | 0.887 (CI 0.849 to 0.926) |
| Gille, 2019 [73] | serum | ECL-based assay | 149 | PLS (11)  PMA (6) | ALS vs PLS | 80.5% (Ci 73.3–86.6) | 90.9% (Ci 58.7–99.8) | 88 pg/ml | 0.89 (Ci 0.83–0.93) |
|  |  |  |  |  | ALS vs PMA | 81.2% (CI 74.0–87.1) | 66.7% (CI 22.3–95.7) | 86 pg/ml | 0.71 (CI 0.63–0.78) |
|  |  |  |  | *Disease controls (82):* GBS (48), CIDP (20)  hSP (14) | ALS vs disease controls (hSP excluded) | not reported | 63.2% (CI 50.7–74.6) | 139 pg/ml | 0.58 (CI 0.51–0.64) |
|  |  |  |  |  | ALS vs hSP | 89.3% (CI 83.1–93.7) | 78.6% (CI 49.2–95.35) | 55 pg/ml | 0.84 (CI 0.78–0.90) |
| Kasai, 2019  [109] | plasma | SiMOA | discovery cohort: 29 | *Non-neurological controls (29)*  *NMD patients (46)* | ALS (discovery cohort) vs controls | not reported | not reported | not reported | 0.6659 |
|  |  |  |  |  | ALS (validation cohort) vs NMD | not reported | not reported | not reported | 0.7824 |
|  | CSF |  | validation cohort: 46 |  | ALS (discovery cohort) vs controls | not reported | not reported | not reported | 0.7206 |
|  |  |  |  |  | ALS (validation cohort) vs NMD | not reported | not reported | not reported | 0.9012 |
| Abu-Rumeileh, 2020  [63] | CSF | ELISA test IBL, HamburgGermany | 80 | *healthy controls (43)*  *ALS mimics (46)* | ALS vs healthy controls | 96.3 % | 97.7 % | 1207 pg/mL | 0.981 ± 0.011 |
|  |  |  |  |  | ALS vs *ALS mimics* | 91.7 % | 91.3% | 1955 pg/ml | 0.922 ± 0.031 |

*Abbreviations*: ALS: amyotrophic Lateral Sclerosis, AUC: area under the curve; CI: confidential interval; NfL: neurofilament light chain; pNfH: neurofilament heavy chain; CSF: cerebrospinal fluid; ECL: elettrochemoluminescence assay; ELISA: enzyme -linked immunoadsorbent assay; MND: motor neuron disorder; fALS: familiar ALS; SiMoA: single-molecule array. FTD: Fronto-Temporal-Dementia; MMN: multifocal motor neuropathy; PLS: primary lateral sclerosis, CIDP: Chronic inflammatory demyelinating polyneuropathy; hSP: hereditary spastic paraplegia, GBS: Guillain-Barre Syndrome; NMD: neuromuscular disease.

**Table 2. Data on longitudinal behaviour of NFs in MND according to current literature**. The table displays a list of studies with at least one longitudinal follow-up after the first observation for the neurofilament of interest; the main findings are highlighted in bold characters. Methods for NF quantification and the potential limits of these findings are briefly reported.

| **Neurofilament isoform** | **Study** | **Biomatrix** | **Methods** | **Concentration range of the kit** | **Concentration range of cross-sectional results** | **Longitudinal sample size (n)** | **Genetic carriers (n)** | **Longitudinal data** | **Potential limits** |
| --- | --- | --- | --- | --- | --- | --- | --- | --- | --- |
| NfL | Lu, 2015  [52] | serum, plasma and CSF | ECL-based assay | Linearity tested  1-50000 pg/mL | IQR plasma: 54.4-158.4 pg/mL  IQR serum: 54.5-151 pg/mL  IQR CSF: 4376-11736 pg/mL | Plasma: 67  Serum: 43  CSF: 24 | excluded | plasma NfL does not change over time  serum NfL have small ns increase over time: **stable NfL levels in blood for 15 mts follow-up**  CSF NfL: small increase in fast and slow progressors | 1) 3 cohorts testing different biomatrices, not related blood and CSF in the same patient.  2) Small sample for CSF collected longitudinally |
|  | Skillback, 2017  [79] | CSF | In-house method [I method] and ELISA kit Uman Diagnostics [II method] (NF-light ELISA kit, UmanDiagnostics AB, Umea°, Sweden); normalization of the two methods | I method: detection limit 250 ng/mL II method: detection limit 125 ng/mL (linearity 125-16,000 ng/mL)  III method: detection limit 50 ng/mL; linear correlation with previous methods but resulted in higher levels of NfL (normalization required) | 970-3600 ng/mL | 69 | unknown | 67% of MND showed **higher CSF NFL concentration at a later stage of disease**, those without rising levels had higher NFL at baseline | 1) use of two different detection methods, the first less sensitive;  2) no notion of the timeline of longitudinal sampling (after how many months the second sample, in which phase of disease, etc) |
|  | Poesen, 2017  [65] | CSF | pNfH: Biovendor, Brno, Czech Republic;  NfL: UmanDiagnostics AB, Umea, Sweden; UD51001 | pNFH: 62.5–4000 pg/ml  NfL: 1-10000 pg/mL | pNFH: 114–18089 pg/mL  NfL: 370–108909 pg/mL | 17 | 26 /220 in cross-sectional cohort  not specified in longitudinal cohort | CSF **pNfH levels rather stable** over time, CSF **NfL increased** over time for a subset of **intermediate and fast disease progressors** | 1) small sample for longitudinal observation;  2) sampling at different disease duration |
|  | Benatar, 2018  [86] | serum | ECL-based assay | reference to previous work [Gaiottino, Plos One 2013]  **15.6-10000 pg/mL** | 21–555 pg/mL | 11 | 3 | **stable levels of NfL** in sporadic ALS | 1) small sample size;  2) follow-up very sparse without knowledge of clinical progression |
|  | Verde, 2019  [64] | serum | SiMOA, time-normalized follow-up NfL levels | 0.686–500 pg /mL [Quanterix] | 14.6 - 908 pg / mL | 29 | 8 / 224 in cross-sectional cohort | **Overall stable**: the variations in NfL from first to second sample ranged from −69.3 to +189 pg/mL (median, −0.1 pg/mL). | small sample size |
|  | Gille, 2019  [73] | serum | ECL-based assay | reference to previous work [Gaiottino, Plos One 2013]  15.6-10000 pg/ml | 0.3 -1141 pg/mL | 16 | 15 / 149 in cross-sectional cohort | **relatively stable over time**, although an increase could be observed within the first 20 months after onset of disease, irrespective of the disease progression rate | 1) small sample for longitudinal observation;  2) sampling at different disease duration;  3) no information of pNfH levels related to clinical outcome |
|  | Benatar, 2020 [81] | serum | SiMOA | 0.686–500 pg /mL [Quanterix] | 2- 369 pg/mL | 106 | 8 | relatively stable over time, computation of longitudinal trajectories by average slope: 0.011 log units/ month (95% CI, −0.054 to 0.076) | well-conducted prospective longitudinal study representative of ALS population; the only potential limit might be sample collection was at some latency from diagnosis (average: 0.7 year), and the study population was skewed towards a slow progression (ALSFRS-r slope: 0.65/month) than general ALS population (1/month, PRO-ACT) |
| pNfH | Lu, 2015  [77] | plasma | in-house ELISA for hyperphosphorylated NfH (NfHSMI34) and variably phosphorylated NfH (NfHSMI35) | linearity tested for serial dilutions with urea pre-analytical tretament to overcome the "hook effect", tested in normalized optical density (NOD) | not reported | 74 | 7 / 136 in cross-sectional cohort | **slight increase** in plasma pNfH for **slow progressors** (32), **no change** for **intermediate** progressors (24), **slight decrease** for **fast progressors** (32) | in-house immunoassay, now better technologies for pNfH quantification |
|  | McCombe, 2015  [110] | serum | ELISA kit EnCor Biotechnology Inc., Gainesville, FL, USA | linearity not tested; from the manufacturer, linearity for standard curve: 0.156 - 10 ng/mL | Not reported | 98 | not reported | **overall rise and then fall** in pNfH levels for ALS pts with at least 3 longitudinal samples; different trajectories according to survival | potentially better immunodetection techniques |
|  | Gendron, 2017  [84] | CSF | Meso Scale Discovery immunoassay with mouse antihuman pNFH anti-body and a sulfo-tagged polyclonal anti-pNFH antibody as capture and detection antibodies | not reported | 50 - 3119 pg/mL non c9-ALS or non c9-ALS-FTD  117 - 4671 pg/mL for c9-ALS or c9-ALS-FTD | 44 | 27 | **no change** in longitudinally collected pNfH in c9ALS, c9ALS-FTD (both groups: 27 pts) and non-c9 ALS (17 pts); no difference between fast and slow progressors | 1) small sample size;  2) no information of pNfH levels related to clinical outcome |
|  | Benatar, 2019  [88] | serum | CE marked ELISA (Euroimmun AG, Lubeck, Germany) | 9.4 - 1000 pg/mL | 639-11418 pg/mL | 16 | 10 | **stable levels** of serum NfH in ALS pts; among mutation carriers who convert there is an  increase in serum pNfH in advance of the appearance  of manifest disease | 1) small sample for longitudinal observation;  2) sampling at different disease duration;  3) no information of pNfH levels related to clinical outcome |
|  | Benatar, 2020  [81] | serum | SiMOA | 0–2,000 pg/mL | 1–976 pg/mL | **106** | **8** | relatively stable over time, computation of longitudinal trajectories by average slope: 0.006 log units/month month (95% CI, −0.063 to 0.084) | well-conducted prospective longitudinal study representative of ALS population; the only potential limit might be sample collection was at some latency from diagnosis (average: 0.7 year), and the study population was skewed towards a slow progression (ALSFRS-r slope: 0.65/month) than general ALS population (1/month, PRO-ACT) |

*Abbreviations*: NfL: neurofilament light chain; pNfH: neurofilament heavy chain; CSF: cerebrospinal fluid; ECL: elettrochemoluminescence assay; ELISA: enzyme -linked immunoadsorbent assay; ns: not significant; MND: motor neuron disorder; mts: months; pts: patients; SiMoA: single-molecule array.


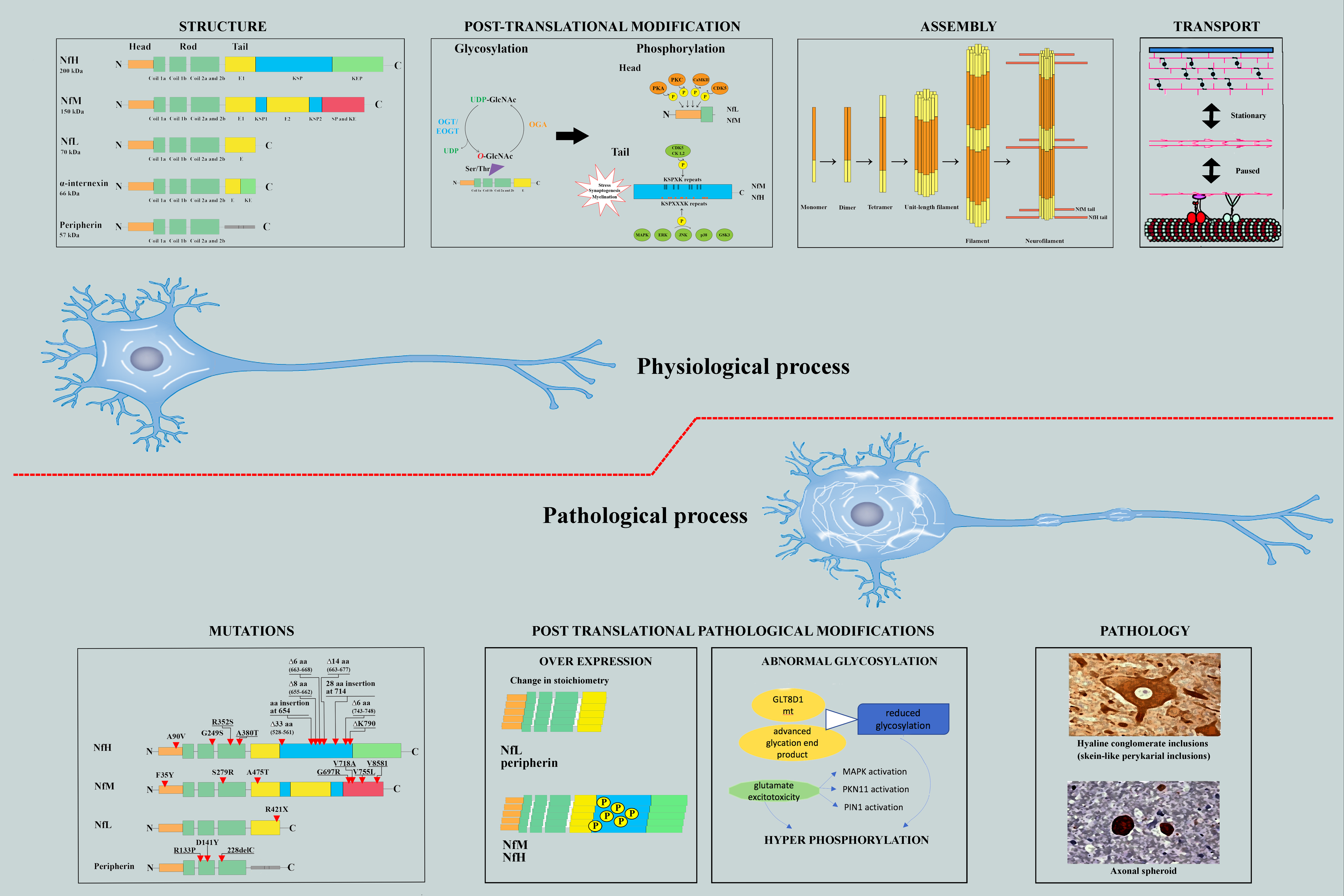


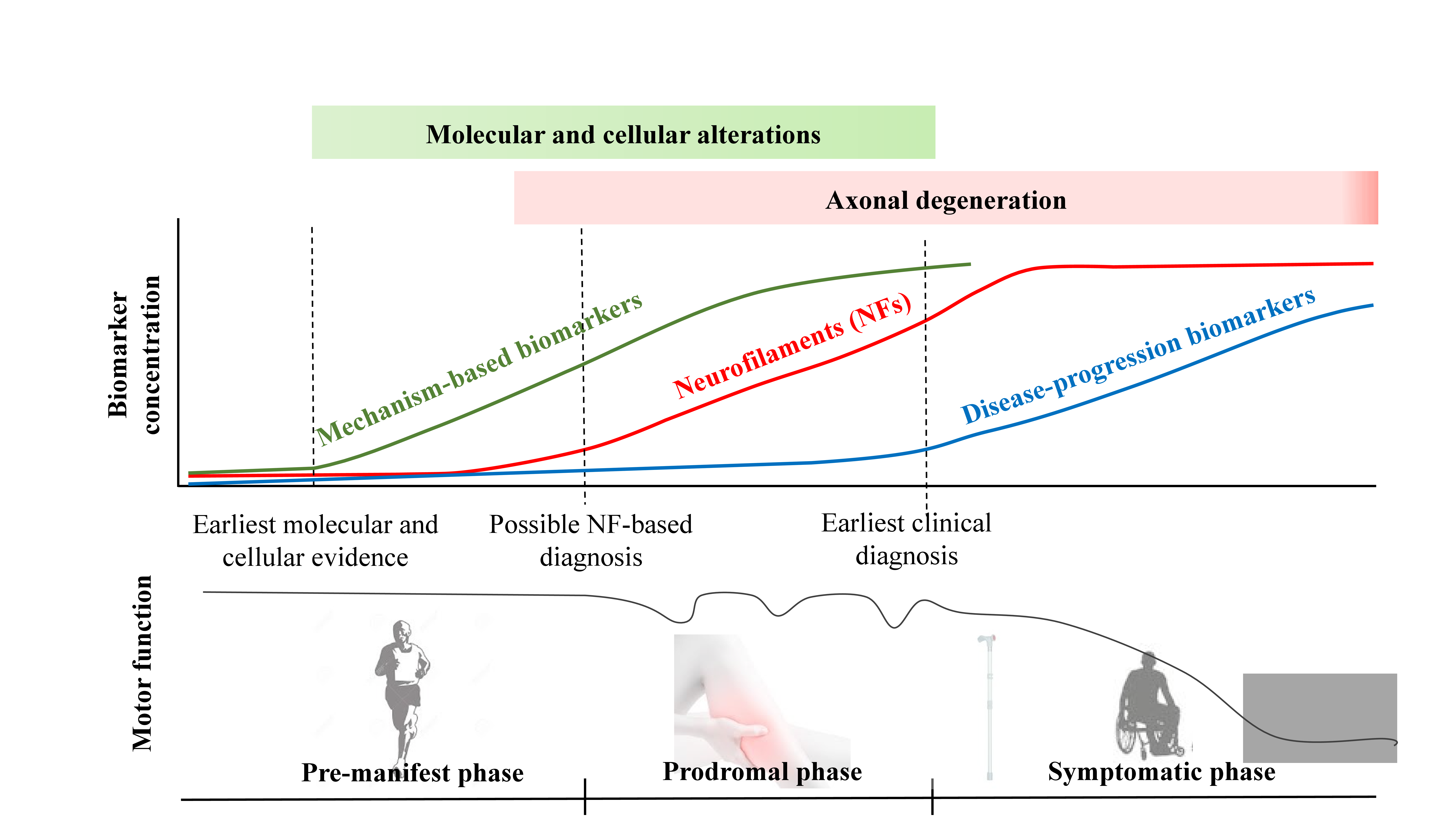


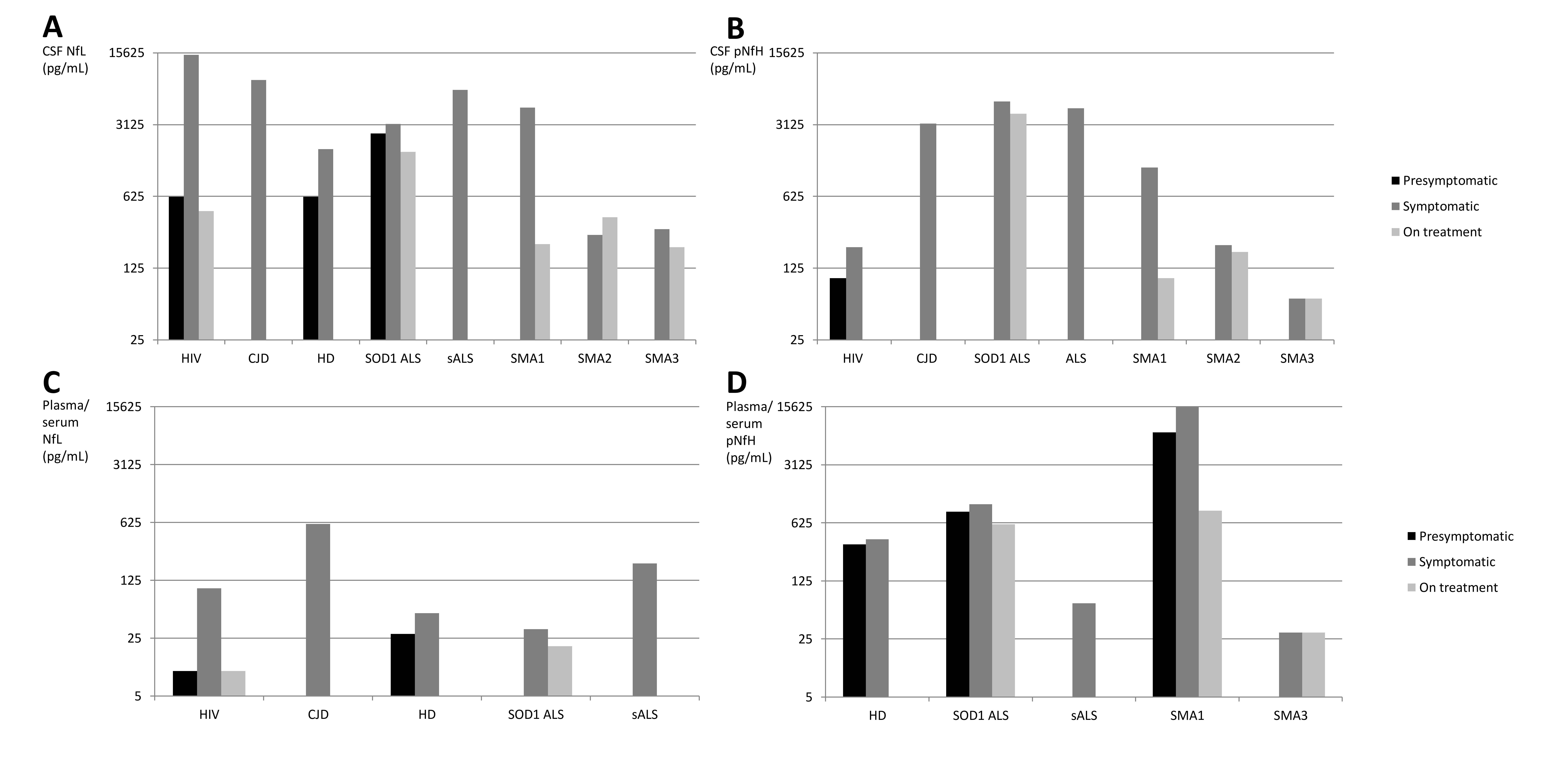

Supplement: Supplementary file 1 — Additional file 1. [file 13024_2020_406_MOESM1_ESM.docx]
